# The nature and nurture of cell heterogeneity: accounting for macrophage gene-environment interactions with single-cell RNA-Seq

**DOI:** 10.1186/s12864-016-3445-0

**Published:** 2017-01-07

**Authors:** Quin F. Wills, Esther Mellado-Gomez, Rory Nolan, Damien Warner, Eshita Sharma, John Broxholme, Benjamin Wright, Helen Lockstone, William James, Mark Lynch, Michael Gonzales, Jay West, Anne Leyrat, Sergi Padilla-Parra, Sarah Filippi, Chris Holmes, Michael D. Moore, Rory Bowden

**Affiliations:** 1 Wellcome Trust Centre for Human Genetics (WTCHG), University of Oxford, Oxford, OX3 7BN UK; 2 Weatherall Institute of Molecular Medicine (WIMM), University of Oxford, Oxford, OX3 9DS UK; 3 Division of Structural Biology, University of Oxford, Oxford, OX3 7BN UK; 4 Sir William Dunn School of Pathology, University of Oxford, Oxford, OX1 3RE UK; 5 Fluidigm Corporation, 7000 Shoreline Ct Ste 100, South San Francisco, CA 94080-7603 USA; 6 Department of Statistics, University of Oxford, Oxford, OX3 3LB UK

**Keywords:** Single-cell sequencing, Single-cell culture, Single-cell imaging, Macrophage heterogeneity, Signaling microenvironment

## Abstract

**Background:**

Single-cell RNA-Seq can be a valuable and unbiased tool to dissect cellular heterogeneity, despite the transcriptome’s limitations in describing higher functional phenotypes and protein events. Perhaps the most important shortfall with transcriptomic ‘snapshots’ of cell populations is that they risk being descriptive, only cataloging heterogeneity at one point in time, and without microenvironmental context. Studying the genetic (‘nature’) and environmental (‘nurture’) modifiers of heterogeneity, and how cell population dynamics unfold over time in response to these modifiers is key when studying highly plastic cells such as macrophages.

**Results:**

We introduce the programmable Polaris™ microfluidic lab-on-chip for single-cell sequencing, which performs live-cell imaging while controlling for the culture microenvironment of each cell. Using gene-edited macrophages we demonstrate how previously unappreciated knockout effects of SAMHD1, such as an altered oxidative stress response, have a large paracrine signaling component. Furthermore, we demonstrate single-cell pathway enrichments for cell cycle arrest and APOBEC3G degradation, both associated with the oxidative stress response and altered proteostasis. Interestingly, SAMHD1 and APOBEC3**G** are both HIV-1 inhibitors (‘restriction factors’), with no known co-regulation.

**Conclusion:**

As single-cell methods continue to mature, so will the ability to move beyond simple ‘snapshots’ of cell populations towards studying the determinants of population dynamics. By combining single-cell culture, live-cell imaging, and single-cell sequencing, we have demonstrated the ability to study cell phenotypes and microenvironmental influences. It’s these microenvironmental components - ignored by standard single-cell workflows - that likely determine how macrophages, for example, react to inflammation and form treatment resistant HIV reservoirs.

**Electronic supplementary material:**

The online version of this article (doi:10.1186/s12864-016-3445-0) contains supplementary material, which is available to authorized users.

## Background

Macrophages - cells that phagocytose microbes, unhealthy and cancerous cells - are at the heart of human aging and pathology from infectious and noninfectious aetiologies. As immune sentinels, macrophages exhibit a variety of pro- and anti-inflammatory phenotypes. At the cellular level these phenotypes are determined not only by (epi-)genetic lineage but are also highly plastic to changing tissue environments [1]. The interplay between these phenotypic drivers underlies many macrophage-mediated pathologies. For example, the complex infectious dynamics between HIV-1 and macrophages within particular tissue niches not only prevents virus eradication in patients on antiretrovirals [2], but is a likely source of low grade neuroinflammation leading to neurocognitive decline [3]. Gene-edited macrophages can be used to study genes with known host-pathogen interactions, but in the absence of genetically tractable blood derived macrophages, macrophages derived from genetically modified pluripotent stem cells (PSCs) provide a suitable alternative model system. These stem cell models have the advantage of reproducibly producing large numbers of edited cells under controlled conditions [4, 5]. However, one challenge with stem cell models is the intersection of biological and technical (stem cell differentiation) heterogeneity that needs to be accounted for, making the case for single-cell sequencing. In addition, with such highly plastic cells it is important to be able to study the context of genetic modifiers, by controlling signaling microenvironments and cell interactions. It’s this context that is crucially lost with many single-cell sequencing approaches, and so a technical goal would be to be able to include the effects of multiple environmental, signaling and intervention variables on cell population phenotypes and dynamics.

**Fig. 1:**
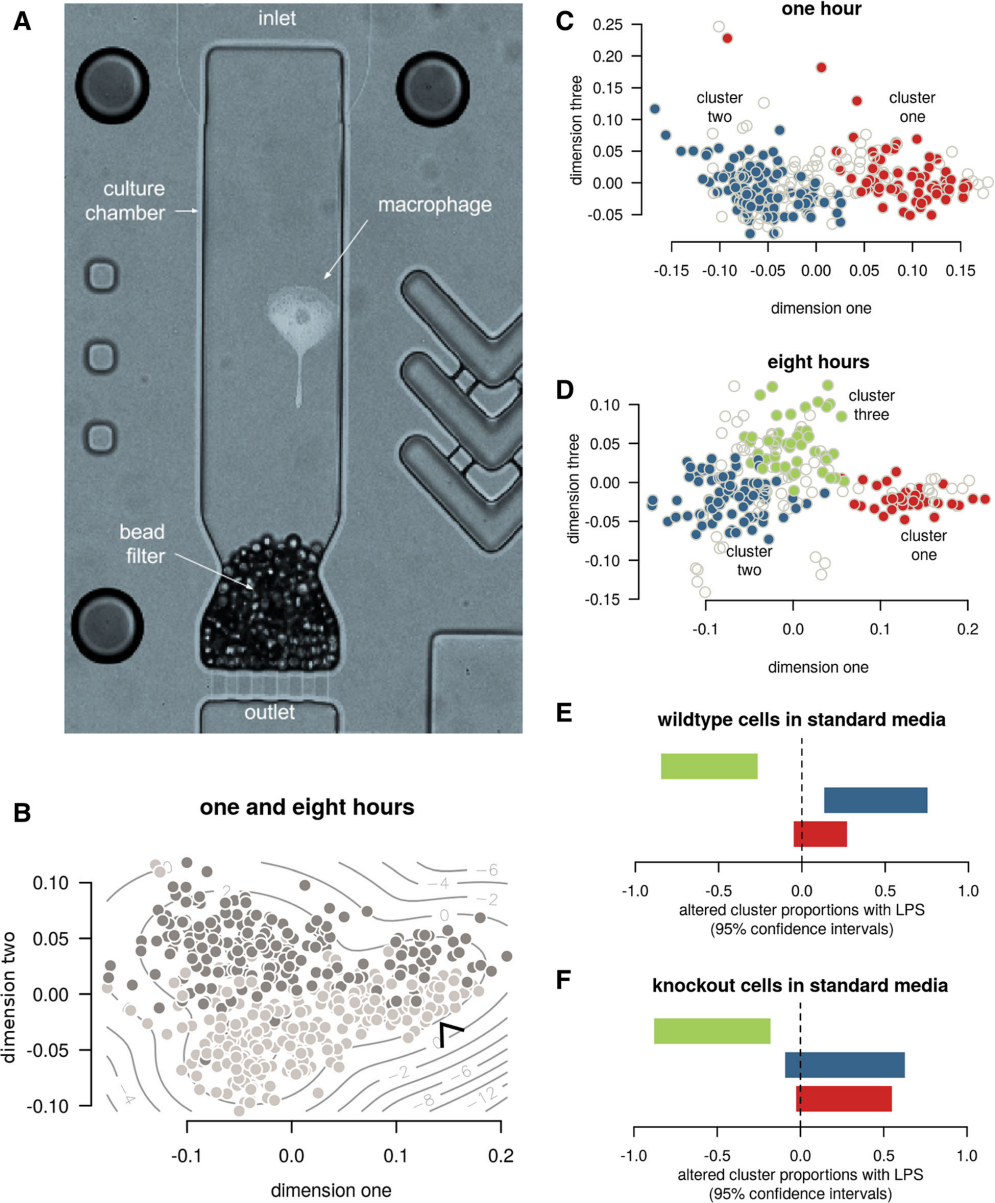
Macrophage culture and subtypes. **a** A single micro-volume culture chamber from the Polaris™ microfluidics chip, containing a macrophage. The media conditions per chamber can be modified to study microenvironmental perturbations. **b** Visualization of the major cell differences — such as with this multi-dimensional scaling of the transcriptomic rank correlations — demonstrated the separation of cells cultured for one hour (light), eight hours (dark), and a reproducible subcluster present across both time points (arrow). **c**&**d** Formal clustering confirmed this subcluster (cluster one) in addition to a third subcluster emerging after eight hours. The inner 50% of cells in each cluster are shown in colour for dimensions one and three to better convey relative cluster positions and densities. **e**&**f** Cluster three significantly reduced its proportion in the context of LPS and standard media. 95% confidence intervals for change in proportions are shown

To allow for a genotype-by-environment investigation we cultured over 500 CRISPR-edited macrophages using a novel microfluidic platform that allows time and dose control over each individual cell’s microenvironment [6] (Fig. 1a). A mixture of wild-type and SAMHD1 knockout monocytes, were generated from HUES-2 human embryonic stem cells [4]. These were differentiated into macrophages that resemble blood monocyte derived macrophages, both phenotypically (high phagocytic ability, expression of CD14, HIV-1 infectability) and transcriptomically [7], while sharing ontogeny with specific tissue-resident macrophages such as microglia of the central nervous system [8, 9]. The need for and importance of such tissue-resident models has recently been reviewed by Sattentau and Stevenson [10]. As the protein of interest in these cells, SAMHD1 is a poorly understood dNTPase that has emerged as a potent HIV-1 restriction factor in non-cycling cells [11]. Its primary physiological role is believed to be the maintenance of genome integrity by limiting the dNTP pool when DNA replication is not required, which is in keeping with observed SAMHD1 downregulation in several cancers [12, 13]. Of direct relevance to innate immunity is its congenital loss of function associated with Aicardi-Goutieres syndrome, a neurodegenerative disease linked to dysregulated inflammation [14]. In order to gain better insights into SAMHD1 biology within macrophages, we set out to study the knockout of this gene in our model. RNA sequencing at a single-cell resolution was necessary to, at a minimum, rule out technical contributions from unwanted cell populations that can occur with imperfect stem cell model differentiation. Furthermore, as SAMHD1 has a direct association with inflammation signaling, we sought to do this in a way that would not only study aspects of inflammatory activation but also cell autonomous effects separate from the influences of macrophage paracrine signaling.

Per microfluidic chip, up to 48 individual macrophages were isolated at random from a mixture of differentially stained wild-type and knockout cells, and cultured in isolation under different inflammatory signaling environments. These culture environments comprised four possible combinations of exposure or non-exposure to lipopolysaccharide (LPS, to stimulate inflammatory activation), and standard or conditioned media. Conditioned medium was derived from bulk macrophage cultures, to simulate the intercellular signaling component that is important in the control of macrophage inflammatory activation. In total this provided eight cell populations per chip (each cell having one of two genetic conditions, and one of four culture environment conditions), with each chip cultured for either one or eight hours in order to account for early temporal changes in response. Details of the experimental work flow are provided in Additional file 1: Figure S2. Cellular phenotypes such as motility and morphology were tracked by live-cell imaging, before the cDNA from cells was harvested for single-cell RNA sequencing. All of the aggregate 16-fold combinations of genotype, environment and time were repeated in at least nine replicate runs to ensure robustness of conclusions. Details of the experimental and imaging methods, including the sequencing quality control are provided in the Methods and Additional file 1.

## Results

### Identifying different macrophage states (phenotypes)

Exploratory RNA-seq data analysis (Fig. 1b) revealed a clear differentiation between one- and eight-hour cultured cells. It also highlighted a subgroup of cells present at both time points that enriched for a gene set previously demonstrated to be differentially expressed in macrophages treated with the (anti-)inflammatory TGFB cytokine (Additional file 1). This provided initial evidence for the potential biological relevance of the observed heterogeneity among the macrophages. To more formally study this latent/emergent (i.e. previously unknown) heterogeneity and the gene expression differences underlying it, we developed a novel hybrid model-driven and non-parametric clustering method constrained to report only cell clusters that were well represented across replicates. We provide further details in the Methods, with all analysis code and results provided in the Additional files 1 and 2 respectively. Our more formal clustering analysis confirmed the existence of the lower-abundance cell state at both time points (called ‘cluster one’ in Fig. 1c&d) but also a reproducible third state (called ‘cluster three’) emerging after eight hours. A search for associations between proportions of the cell states and culture conditions revealed a lower abundance of cluster three when in standard media with LPS (Fisher’s Exact -log_10_P =5.14, Fig. 1e&f), suggesting another inflammatory sub-phenotype in the cells. With these results demonstrating heterogeneity that would otherwise be missed with traditional sequencing, we then asked if any of these cell types resembled the tissue-resident phenotype of interest.

### Identifying the macrophage phenotype of interest

To better understand these latent cell states, we used our modification on the Heskes Rank Product method [15] to estimate upper and lower p-value bounds on differential and heterogeneous gene expression. As detailed in the Methods, we defined differential expression as a global/overall shift in gene expression, while heterogeneous/context-specific expression was defined as highly variable expression across cell states or culture microenvironments. We selected this non-parametric gene ranking approach not only for its focus on result reproducibility, but for its speed and ease of data fusion that make it well suited to single-cell analysis.

**Fig. 2:**
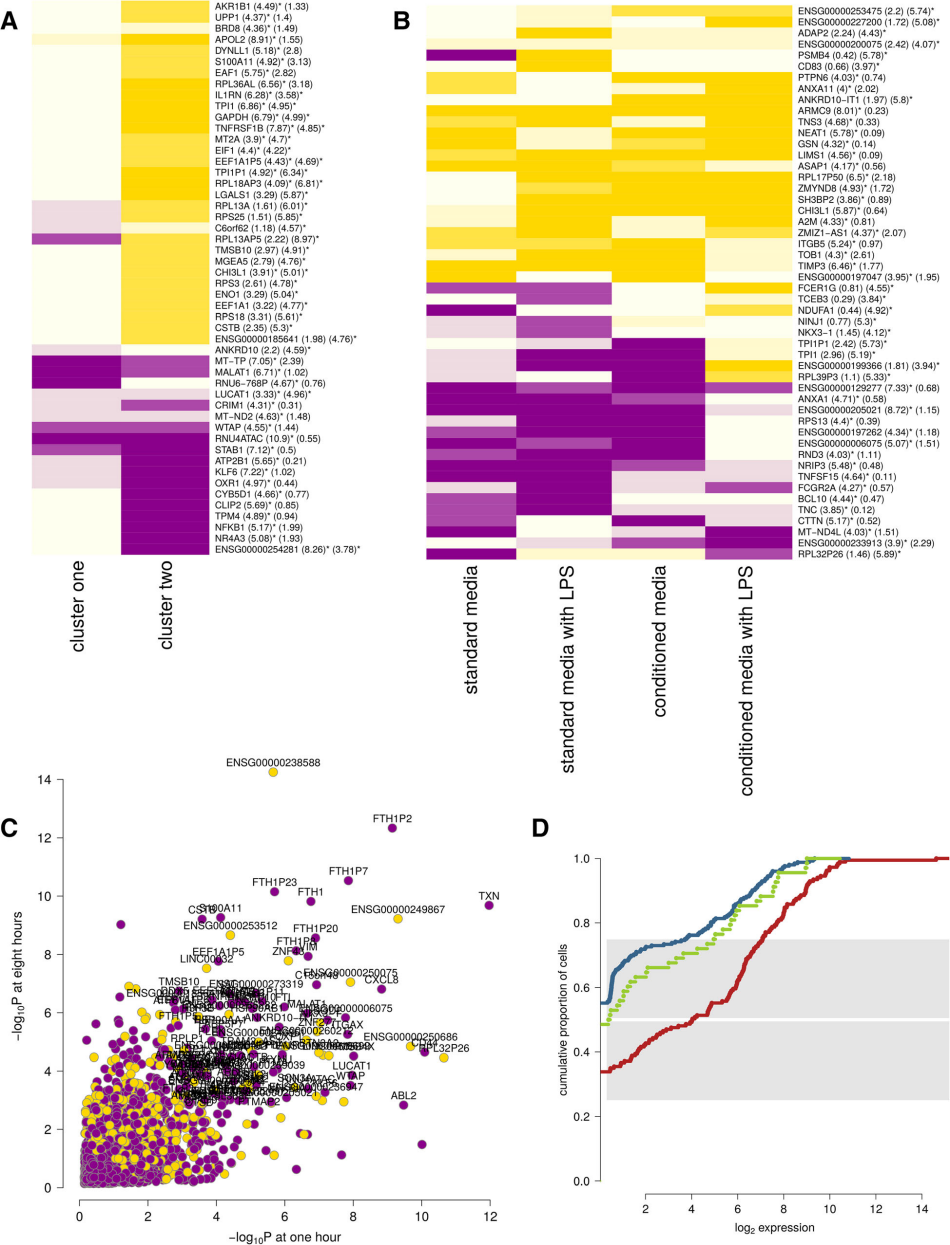
Cell cluster gene expression. In each plot, yellow indicates increased and magenta indicates reduced gene expression. **a**-**b** Heatmaps of the top 50 gene expression results, ranked by statistical significance, are shown for clusters one and two over time (**a**) and cluster three versus other cells, broken down by culture condition (**b**). The numbers provided in parentheses in this and other heatmaps are -log_10_ p-values for differential and heterogeneous (context specific) expression respectively. Results that are globally significant after 5% false discovery rate (FDR) correction are marked with an asterisk. **c** The differential expression results for cluster one versus other cells at one and eight hours. **d** A cumulative proportion plot for *FOXP1* expression broken down by cell clusters. As in other plots, clusters one, two and three are plotted in red, blue and green respectively. Each line plots the cumulative proportion of cells at or below a certain expression level. Cluster one demonstrates greater expression, with approximately half of cluster two and three cells having no detectable expression

Compared against one-hour cultures, most cells in eight-hour cultures (cluster two cells) progressed towards an anti-inflammatory transcriptional signature consistent with the tissue-resident macrophage phenotype of interest. This is evidenced, for example, by the expression of *IL1RN*, encoding IL-1 receptor antagonist, which increased 18-fold over time in cluster two (Fig. 2a). Recombinant IL1RN is used to treat severe inflammatory conditions mediated by its ligand, the archetypal pro-inflammatory cytokine IL-1 [16]. In contrast to other cells, cluster one cells did not move towards a transcriptional profile consistent with a tissue-resident phenotype, and tended to maintain up- or down-regulation of cluster-specific genes. This pattern included lower expression of *GAPDH* and *TPI1*, genes involved in glycolysis, a pathway known to vary across macrophage activation phenotypes [17]. Compared with the other clusters, cluster one expressed higher levels of *FOXP1* across all culture conditions (Fig. 2d, −log_10_P of 6.03 and 4.85 at one and eight hours, both globally significant at a 5% FDR). FOXP1 is a transcription factor involved in maintaining embryonic stem cell pluripotency that must be turned off for complete monocyte differentiation into macrophages [18]. Concerned that selective expression of FOXP1 might represent technical differentiation heterogeneity, we used the imaging data to search for other contributing factors. While we did not find evidence for cell motility or morphology associations, we noted that cluster one cells were more likely to have come into contact with the culture chamber retention beads used to prevent cells from escaping (Fig. 1a, Fisher’s Exact -log_10_P = 4.10). We also noted that cells cultured towards the edges of the chips were more likely to touch retention beads (Fisher’s Exact -log_10_P = 1.79), but that the chip edge positions were enriched for in cluster one, seemingly independent of the bead association (Fisher’s Exact -log_10_P = 3.19, Additional file 1: Figure S6). As cell imaging was performed hourly, this may in part reflect false negatives for detecting bead contact of cells in a less mature and adherent state. Notwithstanding the evidence for both differentiation and environmental factors underlying cluster one, the low correlation between cluster one and off-chip bulk tissue samples (Additional file 1: Figure S7) led us to conclude that it may represent a potentially interesting but low-abundance phenotype that is unrelated to our main question of SAMHD1 biology in tissue-resident macrophages.

A comparison of gene expression in cluster three versus other cells (Fig. 2b) was consistent with a shift towards macrophages with a tissue remodelling phenotype. This was evidenced, for example, by greater expression of *TIMP3*, an inhibitor of extracellular matrix degradation, and *CHI3L1*, a secreted glycoprotein also thought to drive tissue remodelling and a known genetic risk factor for asthma severity [19]. Since cluster three’s transcriptional phenotype most closely resembles that of cluster two (Additional file 1: Figure S18), it seems plausible that this represents an emerging secondary phenotype that requires macrophage signaling when cells are exposed to LPS (Fig. 1e&f). Future studies over longer culture periods will help clarify the microenvironmental dependence and persistence of this cluster.

We focus the remainder of our observations on the main body of cells (cluster two), resembling the desired tissue-resident phenotype. While no cluster two enrichment for particular motility or morphology imaging features were found over these short culture periods, we briefly note an imaging subtype of cells observed to be phagocytosing the retention beads. Phagocytosing macrophages did not assume a distinct transcriptomic cluster 一 likely due to short culture times 一 nevertheless they did enrich for oxidative stress and mitochondrial genes, examples being *PRDX1* and *MT-CO3* (−log_10_P of 4.60 and 4.43 respectively, both globally significant at 5% FDR). This unexpected result suggests an avenue for single-cell studies to explore the temporal dynamics of phagocytosis [20].

### Changes in macrophage behavior (cluster two) with SAMHD1 knockout

**Fig. 3:**
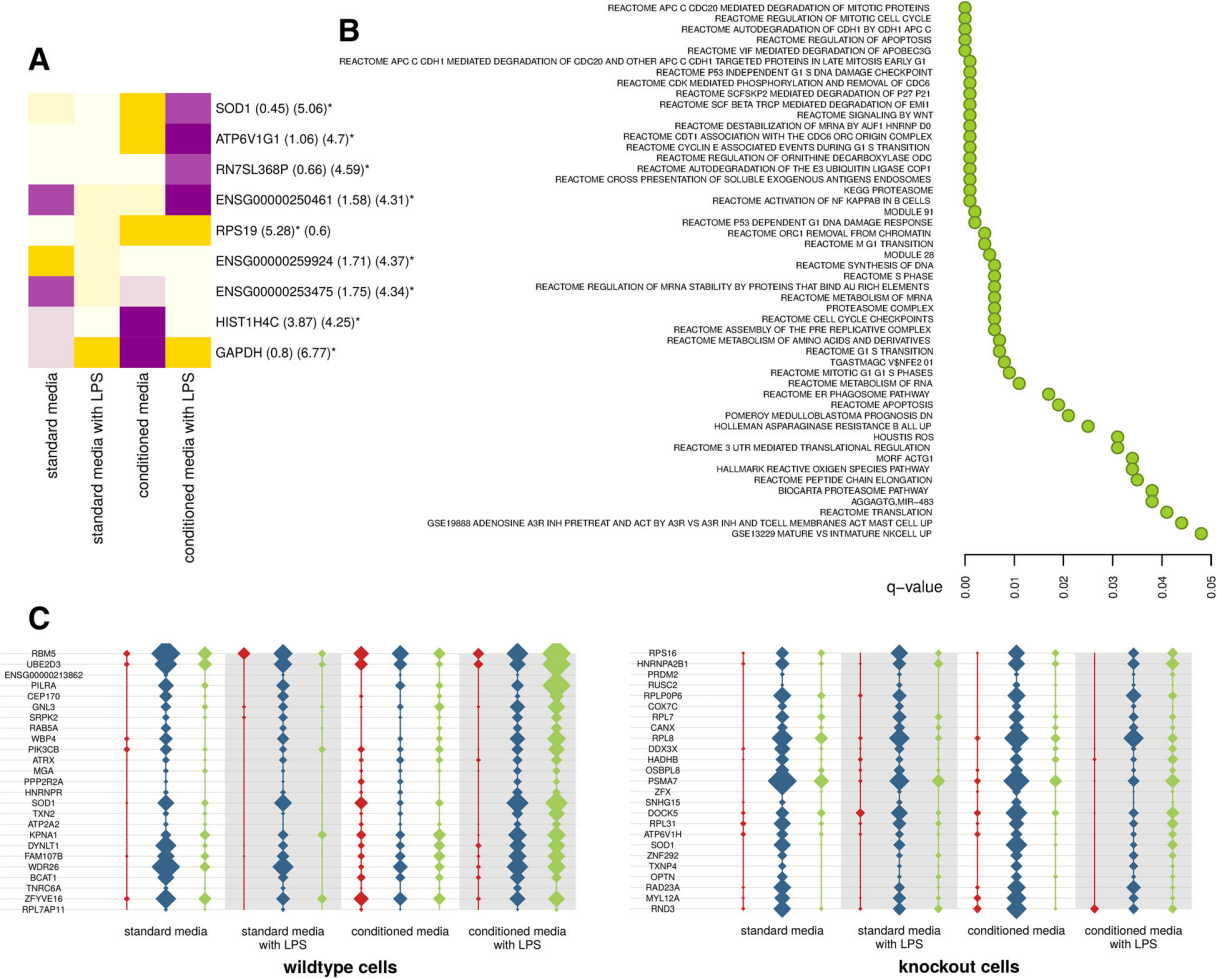
SAMHD1 knockout gene expression (**a**) Heatmap of the difference between knockout and wild-type expression over time, broken down by culture condition. Block colours and numbers in parentheses share the same meaning as in the Fig. 2 heatmaps. **b** Globally significant MSigDB signatures correlating with knockout *SOD1* expression. **c** The expression of *SOD1* and its top co-expressors after eight hours across all cell clusters. Red, blue and green lines correspond to clusters one, two and three respectively. Widths indicate expression level

After filtering our data to focus on the cell subtype of interest (cluster two), we tested for varying knockout and wild-type differential and heterogeneous expression over time. The most striking feature of the globally significant knockout effects was that they were predominantly microenvironment specific (Fig. 3a). Not only does this stress the importance of studying gene-environment interactions in cellular genetics models with known phenotypic plasticity, but in this work allows us to comment on macrophage signaling contributions. Specifically, we note a highly significant association with *SOD1* expression, a gene that encodes the cytosolic isozyme of superoxide dismutase. SOD1 is a superoxide radical scavenger that may confer some protection against HIV-1 neuropathy as part of the oxidative stress response [21], but which has no described associations with HIV-1 restriction factors. As with SAMHD1 loss of function, gain-of-function mutations in SOD1 are associated with neuroinflammation and degeneration, clinically manifesting as amyotrophic lateral sclerosis, which is likely a result of toxic protein aggregates [22]. Overall, *SOD1* transcript levels in this study were similar in wild-type and SAMHD1 knockout cells. However, whereas *SOD1* expression in wild-type cells stimulated with LPS increased over time as expected, expression in knockout cells increased over time in the absence of LPS activation. Not only does this point to a qualitative difference in expression but 一 as can be seen from the barely detectable expression changes in standard media (Fig. 3a) 一 the magnitude of the difference is much greater in conditioned media. This SOD1 oxidative stress response thus has both a notable genetic and signaling component, which may be dampened by identifying and blocking the augmenting signaling factor(s).

Further exploring this oxidative stress response, we noticed a change in *SOD1*’s coexpression (i.e. genes correlating with it) in knockouts versus wild-types (Fig. 3c). Analysing which genes tend to coexpress with *SOD1* allows for biological contextualisation of this effect, as genes are only expressed when required, and so expression correlation implies coregulation. A key difference here being the increased coexpression resolution offered by single-cell analysis to generate gene networks in both perturbed and unperturbed cells, compared with conventional sequencing that is limited to only studying average gene correlations with perturbation. We asked whether there are genes with which *SOD1* alters its rank (Spearman) correlation across cells when SAMHD1 is knocked out. Fig. 3b lists the globally significant signatures from MSigDB v5.1 [23], consisting of gene sets more correlated with *SOD1* in knockout versus wild-type cells. In other words, these highlight genes and pathways coregulating with the altered oxidative stress response in the knockout macrophages. In addition to the expected enrichment for reactive oxygen species (ROS) modulators in this list, we note a striking enrichment for proteasome genes. The proteosome is known to be activated and dysregulated with *SOD1* mutation, leading to aberrant cellular proteostasis [24]. However, it is plausible in these cells that the altered proteostasis is simply a result of the macrophages’ stress response. In particular we note an enrichment for proteasome genes involved with the activation of NF-kappaB (NF-kB), a pro-inflammatory transcription factor on which multiple macrophage signaling pathways converge [25]. A similar proteostasis theme is observed for the regulation of ornithine decarboxylase, a macrophage anti-inflammatory enzyme [26] that metabolises a well described marker of macrophage pro- versus anti-inflammatory phenotype polarisation. With these pathway associations validating this approach by supporting known biology of increased inflammation with SAMHD1 knockout - albeit within the context of oxidative stress and altered proteostasis - we asked if other pathways in Fig. 3b point to unexpected biological themes.

A striking pathway result was the repeated enrichment for proteasome genes involved in the degradation of cell cycle proteins. While the direct causal relationship between cell cycle and SAMHD1 is considered to be via its cyclin-dependent kinase phosphorylation and inhibition [27], these results point to an additional reverse relationship. A tumor suppressor role for SAMHD1 has been proposed based on its maintenance of genome integrity and cancer associations with downregulation [12, 13]. However, the pathways in Fig. 3b counter-intuitively point to p53 dependent and independent G_1_ arrest as part of the DNA damage response. These results highlight a necessary fine balance in SAMHD1 activity in terms of cell cycle control, with too little SAMHD1 arresting cell cycle progression via other tumour suppressors in response to DNA damage. This relationship between SAMHD1 and G_1_ arrest has been demonstrated in dividing fibroblasts that turn senescent [28], though an important difference here is that it suggests the wild-type G_0_ (post-mitotic) macrophages shift to a G_1_ block with SAMHD1 knockout. Finally, and perhaps most interesting in terms of the HIV-1 host-pathogen relationship, is the highly significant pathway enrichment for the degradation of APOBEC3G as part of the altered stress response in SAMHD1 knockouts. This HIV-1 restriction factor causes proviral DNA hypermutation via cytidine deamination, and so shares no known overlap in antiviral activity with SAMHD1 [29].

## Discussion and conclusions

As single-cell methods continue to mature, so will the ability to move beyond simple ‘snapshots’ of cell populations towards studying the determinants of population dynamics. We expect that one area of demand for this type of single-cell functional genomics will be cellular genetics models, with large-scale efforts already underway to generate stem cell banks to support tissue-specific insights into genetic variants [30, 31]. Even with the simplest such models, single-cell sequencing will prove useful to rule out heterogeneity unrelated to the cell phenotypes of interest. In our macrophage model, for example, we have seen that while the majority of cells adopted a tissue-resident phenotype, cells that behave quite differently can be present. As the co-culture and genetic complexity of cellular genetic models increases, so will the demand for methods to confidently map heterogeneity with high replicability across multiple culture or laboratory conditions. Automated and standardized microfluidics present a decided advantage in this regard. In this proof-of-principle work we have been able to generate complex experimental designs within chips over multiple iterations. As all the conditions studied were replicated at least nine times, we have been able to develop and implement statistical models that focus on highly replicated cell behaviors that could easily be integrated with results from other laboratories.

Perhaps the most distinct advantage of such lab-on-chip microfluidics is the ability to perform imaging, temporal and microenvironmental analyses of cell population dynamics. In this study these variables allowed us, for example, to comment on the altered oxidative stress response (*SOD1* expression) with SAMHD1 knockout and macrophage signaling. By blocking the signaling component we observed a significant reduction in this knockout effect. Interestingly, SOD1 and SAMHD1 have known respective gain and loss of function associations with neuroinflammation that are in keeping with effects noticed in this model. We speculate that targeting the observed signaling component may provide an avenue for the treatment of these and other neurodegenerative diseases influenced by innate immunity. Microglia (central nervous system macrophages), for example, are not only implicated in HIV associated neurocognitive disease (HAND), but also in Alzheimer’s disease via aberrant inflammatory signalling [32]. Future work with the Polaris™, assaying single-cell macrophage supernatants, may prove useful in narrowing down this as yet unidentified signaling factor. In this study, single-cell *SOD1* coexpression pathway analysis enriched for altered proteostasis. The most widely observed biological association with this altered proteostasis was a cell cycle (G_1_) block as part of the DNA damage response. While an increased dNTP pool with SAMHD1 loss of function is known to reduce genome integrity, the *SOD1* association with these pathways point to an oxidative stress contribution to DNA breaks triggering G_1_ checkpoint genes. SOD1 may also directly contribute to this arrest via its anti-apoptotic signaling [33]. Perhaps most intriguing is how these associations could provide insights into activating latent viruses within therapeutically intractable reservoirs such as macrophages and resting CD4+ T cells, where SAMHD1 is highly expressed [34]. Triggering apoptosis signaling has been proposed as one therapeutic strategy for HIV-1 activation [35], which would require SOD1 apoptosis inhibition to be minimised. Our knockout cells strongly suppressed *SOD1* expression when activated in conditioned media, suggesting that promoting macrophage activation in combination with SAMHD1 inhibition would, at least *in vitro*, be the most effective strategy to purge latent viruses. Infection of the macrophages with HIV-1 in these microfluidic chips to directly study these effects is one promising approach to study this. Such HIV-1 infection studies with other macrophage knockouts such as the HIV-1 restriction factor APOBEC3G, may prove particularly enlightening, as results from this study suggest a previously unappreciated connection between SAMHD1 loss of function and APOBEC3G degradation. Understanding the conditions under which SAMHD1 inhibition also results in reduced APOBEC3G levels would be of direct relevance to therapies aimed at viral activation. Under these situations, SAMHD1 inhibition might have the desired activation response, but reduced APOBEC3G would enhance the ability of the activated viruses to reinfect other cells.

## Methods

### Stem cells, generation of macrophages and experimental media

The human embryonic stem cell line HUES-2 was obtained from the HUES Facility, University of Harvard [36]. Feeder-free PSC cells were cultured in mTeSR^TM^-1 medium (Stem Cell Technologies) on Matrigel (Corning)-coated tissue culture dishes, passaged with TrypLE (Invitrogen) with the addition of 10 μmol/L Rho-kinase inhibitor Y-27632 (Abcam). A double-nicking CRISPR-Cas9 approach was used to generate SAMHD1-knockout stem cell lines [37]. Plasmid pX462 (gift from Feng Zhang; Addgene plasmids cat. 48141, [38]), expressing the guide RNA, D10A-mutated Cas9 and a puromycin-selection cassette was adapted to target *SAMHD1* at exon 4 (GTGTATCAATGATTCGGACGAGG and CGATACATCAAACAGCTGGGAGG; PAM underlined) or exon 5 target sites (CGTTCACTTATCTGCAGCTCTGG and GGATGTCTAGTTCACGCACTGGG; PAM underlined) using protocols previously described [38, 39]. PSCs were transfected with all four plasmids targeting SAMHD1 using the Neon® Transfection system (Invitrogen) according to manufacturer’s guidelines (2 × 10^6^ cells electroporated with 15 μg DNA using a 100 μL tip at 1000 V, 40 ms pulse width, one pulse), cultured without antibiotics for 48 h and then for 48 h with selection in 0.4 μg/mL puromycin (Sigma). Single-cell clones were generated by plating transfected PSCs at low density onto mitotically-inactivated mouse embryonic feeder (MEF) cells [40, 41] on gelatin-coated tissue culture plates in stem cell medium (KO-DMEM, 2 mmol/L L-Glutamine, 100 mmol/L nonessential amino acids, 20% serum replacement, and 8 ng/mL FGF2; Invitrogen). Clones with modifications at the SAMHD1 locus were identified by high resolution melt analysis. Sequencing confirmed that clone E2 had an out-of-frame insertion (29 and 49 bp) into each allele of SAMHD1 exon 4 and G9 had an out-of-frame deletion (43 bp) in one allele and an in-frame deletion (39 bp) that deleted the essential allosteric GTP binding site (amino acids 135 to 147) and would alter the catalytic site. Macrophages derived from these clones were screened for SAMHD1 expression by western blotting using a mouse anti-SAMHD1 antibody (clone 2D7, Insight Biotechnology Ltd) and a rabbit anti-GAPDH antibody (Sigma) (Additional file 1: Figure S1).

Feeder-free PSC cells were cultured in mTeSR^TM^-1 medium (Stem Cell Technologies) on Matrigel (Corning)-coated tissue culture dishes, passaged with TrypLE (Invitrogen) with the addition of 10 μmol/L Rho-kinase inhibitor Y-27632 (Abcam). A protocol devised in our laboratory was used to generate macrophages from PSC cultures. Briefly, embryoid bodies were formed using the spin method in AggreWells™800 (Stemcell Technologies) plates, each of which was split into two monocyte factories in T175 tissue culture flasks containing ~150 embryoid bodies. The monocytes released into the supernatant were harvested regularly and plated into 96-well plates at 5 × 10^4^ cells per well in macrophage differentiation medium consisting of XVIVO™15 (Lonza) supplemented with 100 ng/mL M-CSF (Invitrogen), 2 mM glutamax (Invitrogen), 100 U/mL penicillin and 100 μg/mL streptomycin (Invitrogen). Four days after plating the media was replaced with fresh macrophage differentiation media with additional 10% fetal bovine serum (FBS; Invitrogen) and the cells were used on day 7 of differentiation. We used three batches (A, B, G, each corresponding to one AggreWells™800 plate) of wild-type cells and two batches (G9: C, D and E2: E, F) each of the two SAMHD1-knockout clones for the Polaris macrophage stimulation experiments.

Four kinds of media (‘conditioned’ and ‘standard’, with or without LPS) were used in the microfluidic chips. To generate conditioned media, stem cell-derived monocytes were plated at 5 × 10^5^ cells/well in a 12-well tissue culture plate and differentiated for 4 days in macrophage differentiation medium followed by 3 days in the presence of 10% FBS. The medium was then replaced with fresh macrophage medium plus 10% FBS with or without 100 ng/mL LPS (Sigma). “Standard media” were generated by incubation of macrophage differentiation medium plus 10% FBS either with or without 100 ng/ml LPS at 37 °C to simulate incubation of conditioned media with cells. For each kind of medium, after 24 h with LPS/mock stimulation, supernatants were recovered by centrifugation, 0.45 μm-filtered and stored at −80 °C then thawed and clarified by centrifugation at 14,100 rcf for 10 min before use.

### Polaris™ protocols

Polaris runs followed the protocol ‘Using Polaris to Generate Single-Cell cDNA Libraries for mRNA Sequencing’ (PN 101–0082 A1, Fluidigm). Set-up parameters are shown in Table S1. Polaris integrated fluidic circuits (IFCs) were prepared for cell capture by a priming step, during which the capture chambers were also coated with the extracellular matrix compound fibronectin (25 ng/μl, cat. F4759, Sigma-Aldrich) for handling adherent cells, and capture beads (prepared to Fluidigm specifications) were loaded to prevent the release of captured single cells (Fig. 1). Priming was arranged to finish as the cell mix became ready for loading (Additional file 1: Figure S2A).

For each experimental run, one well of cells from a single batch each of wild-type and knockout cells was used. One sample was stained with CellTracker™ Orange CMRA Dye and the other with both CellTracker™ Orange CMRA and CellTracker™ Green CMFDA Dye (cat. C34551 and C7025, Thermo Fisher Scientific) in Wash Buffer (Fluidigm). Dye concentrations were adjusted upwards between runs during the experiment to improve sensitivity and resolution: 1 μM for runs 1 and 2; 2 μM for runs 3 to 12; and 3 μM for runs 13 to 20. Wild-type and knockout cells were dual- or single-stained in approximately equal numbers of experiments (Additional file 1: Figure SB). The culture medium was removed and the cells were washed twice with 250 μl Wash Buffer (Fluidigm) then stained with 100 μl of staining solution at 37 °C for 15 min before a further wash with 150 μl of Wash Buffer. The Wash Buffer was removed, 100 μl of 0.5 mM EDTA (cat. 15575, Gibco) in PBS was added and the cells were incubated at 37 °C for 15 min before the EDTA was removed and the cells were resuspended gently in 50 μl of Feed Media (X-VIVO™ 15 cat. BE02-061Q (Lonza), 1% Penicillin-Streptomycin (10,000 U/mL), cat. 15140–122, (Gibco)). Cells were counted on a TC-20TM Automated Cell Counter (Bio-Rad) and samples were adjusted to a final concentration of 350–400 cells/μl.

Fluidigm guidelines (Fluidigm Single-Cell Preparation Guide, PN 100–7697) were used to establish optimal buoyancy at an 4:1 (cells:cell suspension reagent) ratio for the Polaris experiment. For each run, 15 μl each of differentially stained wild type and knockout cells were mixed with 7.5 μl of Cell Suspension Reagent (Fluidigm, PN 101–0434) 25 μl of the resulting suspension, containing an estimated 7000–8000 cells, was loaded on the Polaris IFC. On-board imaging settings to control automatic cell selection were set for each run, with a threshold that varied between 4000 and 6000 for a constant 1.0 s exposure. After completion of the cell selection step, the IFC was removed from the Polaris system and placed in an incubator at 37 °C and 5% CO_2_ for 2 h to allow the cells to settle prior to dosing. Dosing culture media were centrifuged in small volumes (500 μl) at 14,100 rcf for 10 min before 27 μl of medium and 200 μl of Feed Media were loaded into the appropriate wells of the IFC (Additional file 1: Figure S2C). For 1-h dosing runs, the dosing step was stopped manually and for 8-h dosing runs, cells were also dosed at 4 h. Successive runs were scheduled to alternate between one and eight hour dosing.

The Polaris acquired images of all 48 chambers in all available fluorescence channels during cell capture, at the start of dosing and every 1 h thereafter until the end of the run. IFCs were removed from the Polaris for additional, high-resolution imaging on a Leica TCS SP8 confocal microscope (Leica Microsystems) at 3 stages of each run: after cell capture, after the 2-h incubation and at the end of the dosing step, just before lysis. (Additional file 1: Figure S3A). The imaging protocol was designed to acquire bright-field and fluorescence images, for CellTracker Orange and CellTracker Green: 488 nm and 561 nm excitation and hybrid detectors for emission at 500–550 nm and 571–630 nm respectively, were used with a 20× objective and a template to automatically locate the 48 IFC cell isolation chambers, in an image acquisition process lasting ~5 min during which the microscope chamber temperature was maintained at 37 °C. The black vinyl film on the lower surface of the Polaris chip was removed temporarily for each imaging stage. For each time-point and channel, the Polaris stored a single image of the IFC’s 48 cell chambers, 5200 × 1000 pixels in size, in which each pixel had dimensions of approximately 5.5 × 5.5 μm. On the Leica, one image of 512 × 512 pixels, each 1.39 × 1.39 pixels, was captured per cell chamber in bright field, orange and green channels. The initial aims of the image analysis were to differentiate double- and single-stained cells (wild-type and knockout or vice-versa, depending on the run) and to assess cell shape, motility and phagocytosis behaviour. The R package EBImage [42] was used with a set of bespoke R functions to automatically identify individual cell chambers and the cells they carried. Formally, within the large Polaris image(s), cells were detected as clusters of 4 or more of the brightest 30 pixels in the trimmed image corresponding to each cell chamber, for each fluorescent channel. Motility was assessed by measuring changes in the cluster positions between imaging time-points. To assess cell morphology (*circularity*, the proportion of pixels within the smallest circle encompassing the cluster that belong to the cluster), the higher-resolution Leica images were analysed using a re-scaled version of the same algorithm, requiring clusters of 60 or more of the brightest 411 pixels. Automated imaging assessments were checked extensively by eye, leading to the identification of irregularly shaped or unevenly stained cells. Macrophage phagocytosis of capture beads was assessed manually.

For each Polaris run, a bulk control sample of the wild-type and of the knockout cells used in the run was prepared for sequencing. Aliquots containing 2000–4000 of the stained cells loaded on the Polaris were kept at room temperature, incubated along with the loaded IFC for the 2-h incubation step, and kept at 37 °C during dosing. Within 30 min of starting the Polaris lysis step, bulk-cell samples were lysed and processed using the RNA extraction kit RNeasy Micro Kit (cat# 74004, Qiagen) and eluted in 14 μl of RNAse-free water. At the end of the dosing protocol and after any additional imaging, cells were lysed for reverse transcription and amplification for cDNA generation in the Polaris. Master mixes for this procedure were prepared using the SMARTer Ultra Low RNA Kit (Clontech), according to the Fluidigm Polaris protocol with minor modifications. The cell lysis mix (28 μl) contained 8.0 μl Polaris Lysis Reagent, 9.6 μl of a 1/200 dilution of Polaris Lysis Plus Reagent (in PCR-grade water, prepared immediately before use), 9.0 μl SMARTer kit 3′ SMART CDS Primer IIA and (for runs 1–11) 1.4 μl of diluted ERCC spike-in RNA, prepared by adding 1.0 μl of a 1/10 dilution of ERCC ExFold RNA Spike-In Mixes (cat. 4456739, Ambion) to 96.5 μl Polaris Loading Reagent and 2.5 μl SMARTer Kit RNase Inhibitor (40 U/μl). The reverse transcription reaction mix (48 μl) contained 15.5 μl 5× First-Strand Buffer (RNase-free), 1.9 μl DTT, 7.7 μl dNTP mix, 7.7 μl IIA Oligonucleotide, 1.9 μl RNAse Inhibitor, 7.7 μl SMARTscribe Reverse Transcriptase (all SMARTer Kit, Clontech), 2.4 μl Polaris Loading Reagent and 3.2 μl Polaris RT Plus Reagent. The PCR mix (90 μl) contained 63.5 μl PCR-grade water, 10.0 μl 10× Advantage 2 PCR Buffer (not SA – Short Amplicon), 4.0 μl 50× dNTP Mix, 4.0 μl 50× Advantage 2 Polymerase Mix (all Advantage 2 Kit, Clontech) and 4.0 μl SMARTer IS PCR primer. The cDNA products were harvested into a 96-well plate in the arrangement shown in Additional file 1: Figure S2D. Harvest wells with atypical volumes (some with no material, others with an excess) were excluded from further analysis. Bulk control samples comprising 1 μl of RNA extracted from the bulk samples above were each mixed with 4.5 μl of cell lysis mix and processed using the following temperature sequence: 37 °C for 5 min, 72 °C for 3 min, 25 °C for 1 min, then 4 °C (hold), then reverse transcribed using 9.0 μl of the reaction mix, at 42 °C for 90 min followed by enzyme inactivation at 70 °C for 10 min, then 4 °C (hold). Bulk-sample PCRs contained 1 μl of cDNA generated in the previous step and 9.3 μl PCR mix. PCR conditions were as follows: 95 °C for 1 min then 5 cycles of {95 °C/20 s, 58 °C/4 min, 68 °C/6 min}, 9 cycles of {95 °C/20 s, 64 °C/30 s, 68 °C/6 min}, 7 cycles of {95 °C/30 s, 64 °C/30 s, 68 °C/7 min}, then 72 °C for 10 min and 4 °C (hold). After Quant-iT™ PicoGreen (Thermo Fisher) normalization of cDNAs to 0.22 ng/μl, Illumina sequencing libraries were prepared together using Nextera XT DNA Library Preparation Kit (Illumina), according to the manufacturer’s specifications, at quarter-scale on a Beckman-Coulter FXp automated liquid handling instrument, and pooled as up to 192 multiplexed libraries using in-house dual-indexing library tags. Some samples that had failed because of empty cell chambers or the presence of more than one cell in a chamber were included, and two bulk-cell samples per run were included in each pool, which was sequenced as a single lane of 75 b paired-end reads on an Illumina HiSeq 4000 instrument in 4 different runs.

### RNA-Seq data generation and initial quality control

Samples were prepared and paired-end sequenced using the Illumina HiSeq™4000 Sequencing platform, as described above. RNA-seq reads were trimmed for Nextera/Illumina adapter sequences using skewer-v0.1.125 [43]. Trimmed reads were mapped to a modified reference genome comprising the human genome, Homo sapiens GRCh37, and fasta sequences for ERCC spike-ins (ThermoFisher). Reads in gzipped fastq format were aligned using Hisat2 version-2.0.0-beta [44] with default parameters. Duplicate reads were marked using MarkDuplicates.jar implemented in Picard tools v1.92. BAM alignments were name sorted with Samtools version 1.1. Alignment metrics were calculated using CollectRnaSeqMetrics.jar implemented in Picard tools v1.92 for full BAM files and with potential PCR duplicates marked. RNA-SeQC [45] was used to calculate sequencing bias, as the median estimators in Picard can result in zero estimates. Reads mapping uniquely to genes annotated in ENSEMBL release 76 were counted using featureCounts [46] implemented in subread-v1.5.0 [47]. Read distribution between various features - assigned reads (mapped uniquely to exons), multiple mapping, ambiguous mapping, No features (mapped uniquely to intronic and intergenic regions) – was obtained from featureCounts results. Read counts were normalized to Transcripts per million (TPM), and number of detected genes per sample were calculated by counting genes with at least 1 TPM. Details of the supplied data and meta-data provided are provided in Additional file 1, including further QC using the R scater package [48]. In brief, we excluded culture chambers with visually confirmed doublets (two cells), numbers of detected genes more similar to bulk controls, and cells with very low starting cDNA. FASTQ files for the data have been uploaded to the Gene Expression Omnibus (GSE87849).

### Clustering the macrophages and gene expression modeling

All modeling and analyses were performed in the R environment (version 3.2.1, x86_64-pc-linux-gnu 64-bit, Ubuntu 14.04.2 LTS). Multidimensional scaling (MDS) on the cell rank correlations - without gene weighting - was used to reduce one hour and eight hour cells to five dimensions, as detailed in the provided Additional file 1 code wrapped in the cellStates() function. Inclusion of a greater number of dimensions did not influence the clustering. Additional file 1: Figure S16 & S17 demonstrate the cell densities and individual cells for the five dimensions in the eight hour cells, where some clusters of cells can be seen to be unique to individual chips. To ensure reproducibility, the constraint was added that a cluster with fewer than three cells in more than half of the replicates not be considered in the downstream modelling. While such groups of cells may represent illuminating features in the macrophage model’s behaviour, these are not reproducible effects.

Extracting major cell states was performed using a hybrid of model-driven clustering (Gaussian mixtures) and non-parametric clustering (partitioning around medoids), as detailed in the Additional file 1 code wrapped in the consensusCluster() function. Repeated fitting of mixtures to the data - with one chip replicate omitted per iteration - was used to produce a consensus matrix of the proportion of iterations cells share clusters. This was non-parametrically re-clustered, selecting the maximum number of clusters for which experimental replication was strongly represented in each cluster. We note that although this combination of methods was employed, other tested approaches such as hierarchically clustering the consensus matrix produced similar results due to the consensus and reproducibility constraints. The defined clusters are recorded in the cell meta-data (Additional file 2). The major latent cell states identified with exploratory data analysis remain the only two identified major states in the one hour cells, with a third reproducible cluster emerging at eight hours that shares properties with the main group of cells (Additional file 1: Figure S18).

Altered gene expression was modelled as the change in mean conditioned on (i.e. tested per) cell subtype of interest. Overall mean across cell subtypes (μ) was used as a measure of global shift in gene expression, while mean absolute deviation (MAD) of the subtypes was used as a measure of variability and so context/subtype specificity. For example, for d_i_ = change in mean expression over time for cell subtype *i*, μ = (d_1_ + d_2_ + .. + d_n_)/n and MAD = (|d_1_-μ| + |d_2_-μ| + .. + |d_n_-μ|)/n. More robust estimators, such as the use of quantiles, provided similar top hits, so here we present μ and MAD estimates, focusing rather on a rank product framework to determine statistical significance of rank reproducibility per sequencing library [15]. A focus on gene ranks has several advantages well suited to single-cell work: it is non-parametric, robust, comments directly on reproducibility, and allows data fusion or meta-analysis without the need for complex data normalisations. For computational speed, the Heskes rank product algorithm was used to assess bounds on the statistical significance. Reported p-values are the geometric means of the upper and lower bounds provided by the rankprodbounds() function, with the qvalue package used to estimate global significance at 5% false discovery rate [49]. Rank (Spearman) correlations were used to estimate gene co-expression, followed by testing for co-expression signature enrichment with Preranked Gene Set Enrichment Analysis (default settings) [23].

## Additional files

Additional file 1: Supplementary Methods, Figures and Tables. (DOCX 3991 kb)
Additional file 2: Supplementary Data. (XLSX 30285 kb)

## Abbreviations

HAND: HIV associated neurocognitive disease
LPS: Lipopolysaccharide
PSC: Pluripotent stem cells
ROS: Reactive oxygen species
