# Supplementary material for: The nature and nurture of cell heterogeneity: accounting for macrophage gene-environment interactions with single-cell RNA-Seq
Source: BMC Genomics. 2017 Jan 7;18:53. doi: 10.1186/s12864-016-3445-0 (PMC5219790; doi:10.1186/s12864-016-3445-0)
Supplement: Additional file 1: — Supplementary Methods, Figures and Tables. (DOCX 3991 kb) [file 12864_2016_3445_MOESM1_ESM.docx]

# Supplementary Methods

**DATA QUALITY CONTROL, EXPLORATORY DATA ANALYSIS, AND MODELING**

RNA-Seq TPMs and sample meta-data are provided in the Supplemental Data. The provided meta-data per cell are:

1. *chip:* Chip on which the cell was cultured, imaged, and its cDNA harvested (numbered 2 to 20, chip 1 failed harvesting).
2. *chip culture site and chip culture group:* The cell's chip position and grouping (8 groups of 6 cells each per chip). Two cell groups per chip received one of four culture media, as described above. All cells were cultured in isolation, without mixing of incoming or outgoing media.
3. *harvesting plate and harvesting well:* Plates and well positions of harvested cDNA.
4. *sequencing library:* Cells were sequenced in five multiplexed libraries of up to 192 cells each.
5. *ercc spikeins used:* Chips 2 to 11 were run with ERCC spike-ins. Spike-ins were omitted from the remaining runs due to low starting material recovered from the macrophages.
6. *macrophage batch:* The post-mitotic macrophages were differentiated as various embryonic stem cell (ESC) batches, as discussed above.
7. *macrophage samhd1:* Approximately half of the sequenced macrophages were one of two types of *SAMHD1* knockout. Homozygous out-of-frame knockout cells are annotated as 'knockouts', while those annotated as 'dNTPase knockouts' are allosteric knockouts as described above.
8. *macrophage culture time in hours and macrophage culture media:* The macrophage culture times and conditions, as described above.
9. *macrophage staining and macrophage dye concentration in μM:* Dyes and concentrations used for cell imaging.
10. *macrophage within beads, max macrophage displacement per hour, min macrophage circularity, max macrophage circularity, and phagocytosing beads:* Imaging characteristics of the macrophages, as described above.
11. *cDNA in ng per μl:* cDNA harvested per cell.
12. *reads mapping uniquely to ensembl genes to pct reads from mitochondrial genes:* Eight sequence mapping and count variables are provided per cell.
13. *latent spatial:* Exploratory data analysis identified a reproducible association with beads and culture position. This is summarised here as a standardised linear latent variable (first dimension of the multidimensional scaling described in below, Fig. S14).
14. *latent temporal:* A second effect noted with exploratory data analysis was the separation of one hour and eight hour cells. This is summarised here as a standardised linear latent variable (second dimension of the multidimensional scaling described in below, Supplemental Fig. 14).
15. *latent cluster:* Consensus mixture models were used to identify latent cell states in higher dimensions. Latent states were extracted separately for the one hour and eight hour macrophages.

Per chip, empty cell culture chambers were sequenced as negative controls, in addition to sequencing bulk positive controls of a few thousand cells each. Individual macrophages (assessed by imaging) were then further filtered to remove cells with:

1. Likely low starting material (less than 0.1 ng/μl cDNA or log_10_ total gene counts below negative control median)
2. Possible undetected additional cells (log_10_ total gene counts above positive control median)

Any chip harvesting wells with unusually low or high volumes were also excluded from further analysis. As the study benefited from a high number of replicates, this was used as the gene expression filter criterion. Genes with detectable reads in at least half of the replicates are provided with the Supplemental Data, with analysis focusing on genes with detectable expression in at least 80% of replicates. 80% was selected, as a more stringent threshold omitted *SAMHD1*. Overall this provided a median of 1.3 million reads per cell, and 4120 genes with detectable expression in at least 80% of replicates.

Fig. S3 provides summaries of the detected genes, showing the largest proportion of genes to be protein coding, with the top 50 genes (by TPMs) enriching for housekeeper and mitochondrial genes, in addition to macrophage biology. Within the top 50 genes in wildtype cells, those with known macrophage and inflammation biology included: *SPP1* (*IFNG* and *IL-12* stimulating cytokine), *ITGAX* (monocyte fibrinogen receptor), *SAT1* (arginine metabolism), *TXN* (response to intracellular nitric oxide) *CXCL8* (major inflammatory chemokine), *SOD2* (breaks down superoxide anion radicals), *CTSB* (degrades amyloid precursor protein), and *HMOX1* (anti-inflammatory) .

Various dimension reduction visualisations were employed as part of the exploratory data analysis to better account for technical associations in the data. Fig. S4 provides a principal components analysis (PCA) plot of cells, using the 500 most variably expressed genes across the macrophages and controls. As is typical of scRNA-seq data, the total number of detected genes per cell is a dominant variance component, with positive controls forming the yellow cluster and negative controls clustering towards the opposite dark blue end. A second cluster of cells that does not fit with the gradient is evident in the third component.

Removing the controls emphasises the sub-cluster, a group of cells with a greater proportion of mitochondrial reads and slightly lower cDNA recovery (Fig. S5). While this might ordinarily flag potentially apoptotic and degraded samples, this would not be in keeping with the high gene counts or unaltered 3'UTR to 5'UTR coverage bias. Annotating the PCA by chip and macrophage position on chip highlighted associations with the sub-cluster, taken as cells with component one value greater than 25 (Fig. S6). The most notable cluster association was within-chip cell position. Cells with low or high culture site numbers (cells at the edges of a chip) were found to be about three times as likely to occur in the sub-cluster than any other cell. As shown in Fig. S6 the cells towards the centre of the chip express higher *HIF1A* and *TNF*. *HIF1A*, a transcription factor induced by hypoxic conditions, is also induced as part of the inflammatory response. As shown in Fig. S6, a *HIF1A* gradient is not seen in reference K562 cells, and greater hypoxia towards the centre of chips does not fully explain chips 4 and 13. Nor does it explain the greater correlation between the main cluster of cells and the bulk controls (Fig. S7). Fig. S8 plots the sequencing bias as a function of chip position, showing no evidence of sample degradation.

The within-chip effect is partially confounded with the cDNA harvesting plate and well position (Fig. S9), though harvesting effects do not explain the altered gene expression related to macrophage activation or the bead contact associations (discussed below). No clustering association was observed with sequencing library or the inclusion of ERCC spike-ins. Spike-ins were included for the first half of the study, but later omitted due to low starting material from the macrophages. Annotating the cells by culture batch, knockout status, culture time and conditions demonstrated a clear separation of cells by time (Fig. S10), which corresponds with the gradient of total detected genes per cell (Fig. S4).

The macrophage batches recorded are differentiation batches of *SAMHD1* wildtype (WTA, WTB, WTG) or knockouts (KOC, KOD, KOE, KOF). Batches KOE and KOF are of a homozygous out-of-frame deletion with no detectable protein and low levels of transcript (Fig. S11), while KOC and KOD have one allele as an in-frame deletion of an allosteric GTP binding site and the other as an out-of-frame knockout. Batches KOC and KOD are referred to as the dNTPAse knockout in the cell meta-data. Protein expression is shown in Fig. S1.

Chip culture chambers each contained a retention bead filter, with 76% of the macrophages embedding under, at some point migrating against, or phagocytosing the beads. While not clear in Fig. S12, macrophages not at the edges and not interacting with beads were less likely to occur in the sub-cluster. This is formally tested as part of the cluster enrichment analysis. No sub-cluster association could be found with macrophage dyes.

Examples of three alternative dimension reduction techniques are provided in Figs. S13 & S14, demonstrating that the sub-cluster effect in these. This effect is possibly best summarised by the multidimensional scaling (MDS) on the Spearman correlation distance of cells (Fig. S14), where the separation of one hour and eight hour cultured macrophages is maintained in both clusters. The two dimensions in Fig. S14 have been provided in the sample meta-data as latent_spatial and latent_temporal respectively.

Preranked Gene Set Enrichment Analysis, using default settings, significantly (FDR q-values < 0.05) enriched for the following gene sets positively correlated with dimension one (the sub-cluster):

1. Genes with high-CpG-density promoters bearing histone H3 trimethylation mark at K27
2. Genes with lower expression in unperturbed macrophages versus those cultured with TGFB cytokine
3. Genes with greater expression in control versus infected dendritic cells

As there is clear inflammation pathway association maintained at both one and eight hours, this sub-cluster of cells was not filtered but considered as a sub-phenotype for further analysis. Gene sets positively correlated with dimension two (the temporal effect) significantly enriched for:

1. Genes upregulated 1h after TNF exposure
2. Macrophage genes upregulated with LPS

This suggests a temporary activation-like response in cells freshly seeded into the culture chambers, which is further supported by weaker one hour correlations with the bulks (Fig. S7). Fig. S15 provides a summarised version of Fig. S14, demonstrating the time based clustering.

No additional normalisation was applied to the data, except for adjusting TPM calculations in cells with ERCC spike-ins to only consider endogenous genes, such that the measures of gene expression were congruent with the TPMs for cells without spike-ins. Clustering of cell states was performed using the two functions provided below. The first, cellStates() was used to create a reduced dimension representation of the data for clustering using consensusCluster().

## Results from the Rank Product testing of gene expression differences between cells are provided in the Supplemental Data, provided as the following columns:

1. *total logP:* The -log_10_ p-value of the rank product (across sequencing libraries) for the mean response (across micro-environments). P-values were calculated for high and low ranking genes, with the only smallest p-value per gene reported.
2. *total qvalue:* The matching q-values at a 5% false discovery rate correction.
3. *conditional logP:* The -log_10_ rank product p-value on the mean absolute deviation from the mean response, to highlight genes with cell signalling specific responses.
4. *conditional qvalue:* The q-values at a 5% false discovery rate correction.
5. *standard media, standard media with LPS, conditioned media, conditioned media with LPS:* The estimates of response broken down by culture microenvironment. The wildtype and knockout estimates provided in the Supplemental Data are the expression differences over time.

**CODE FOR CLUSTERING CELLS**

##############################################################

*# generate a reduced dimension representation of cell states #*

##############################################################

cellStates <- function( tm, *# tm=1 or tm=8 hour cells*

sb=NULL, *# optional subsample of cells (logical vector)*

wt=NULL ) { *# optional weighting of genes (strictly positive)*

sub <- cellMetadata$macrophage_culture_time_in_hours==tm *# cells to cluster*

if ( !is.null( sb ) ) sub <- sub & sb

if ( is.null( wt ) ) wt <- rep( 1/nrow( tpms ), nrow( tpms ) ) *# gene weighting*

ex <- tpms[, sub] *# expression data subset*

ex <- apply( ex, 2, rank ) *# rank expression for robust cell correlation...*

ex <- cov.wt( ex, wt=wt, cor=T )$cor *# ...with optional weighting*

ex <- data.frame( cmdscale( as.dist( ( 1 - ex )/2 ), 5 ) ) *# multidimensional scaling*

colnames( ex ) <- paste( "dimension", 1:ncol( ex ), sep="_" )

ex

}

###############################################

*# cell states by consensus mixture clustering #*

###############################################

consensusCluster <- function( tm, *# tm=1 or tm=8 hour cells, passed to cellStates()*

wt=NULL, *# optional gene weighting, passed to cellStates()*

dm=1:5 ) { *# cell state dimensions to consider*

require( "mclust" ) *# both available in the CRAN repository*

require( "cluster" )

cstate <- cellStates( tm, NULL, wt )[, dm] *# cell states over all replicates*

rp <- factor( cellMetadata[row.names( cstate ), "chip"] ) *# chip replicates*

cc <- array( 0, *# repeated clustering with one chip replicate omitted per iteration*

dim=c( nrow( cstate ), nrow( cstate ), length( levels( rp ) ) ),

dimnames=list( row.names( cstate ), row.names( cstate ), NULL ) )

for ( i in seq( length( levels( rp ) ) ) ) {

sb <- cellMetadata$chip != levels( rp )[i]

cstateSub <- cellStates( tm, sb, wt )[, dm]

cclustSub <- Mclust( cstateSub, 2:5, modelNames="EII" ) *# mixtures capped at 5*

cclustSub <- cclustSub$classification %*% t( 1/cclustSub$classification ) == 1

cc[colnames( cclustSub ), colnames( cclustSub ), i][cclustSub] <- 1

}

cc <- apply( cc, c( 1, 2 ), mean ) *# proportion of iterations cells share clusters*

cc <- max( cc ) - cc *# as a distance matrix of consensus clustering*

nm <- 2:5 *# number of consensus clusters to consider (capped at 5)*

for ( i in seq( length( nm ) ) ) {

cclust <- pam( cc, nm[i] )$clustering *# nonparametric clustering*

tb <- table( cclust, rp ) *# are chip replicates per cluster...*

tb <- rowSums( tb>=3 ) >= length( levels( rp ) )/2 *# ...well represented?*

if ( !all( tb ) ) nm[i] <- NA *# if not, don't consider in model selection*

}

cstate$consensus <- pam( cc, max( nm, na.rm=T ) )$clustering *# partitioned*

cstate

}

##

# Supplementary Figures and Tables

**Supplemental Table 1.** Polaris set-up

| Feature | Program /Option Selected |
| --- | --- |
| Dose response/Culture Cells Experiment | Dose response |
| Suspension/Adherent Cells Priming | Adherent cells |
| Input volume of cell sample (Cell Selection) | 25 µl (inlet 1) |
| Fluorescence Channels (Cell Selection) | VIC, Threshold 2000, 1.0s exposure |
| Dose response - dosing time | 1h or 8h |
| Post Stain/Wash Step | Wash Only |
| Chemistry | Default (not customizable) |


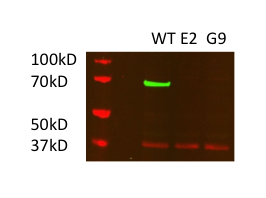


**Fig. S1.** Western blot of SAMHD1-knockout macrophages. SAMHD1 (green) is detectable in the wild-type (WT) sample but not in either knockout clone. Red signal in sample lanes is GAPDH.

**A**


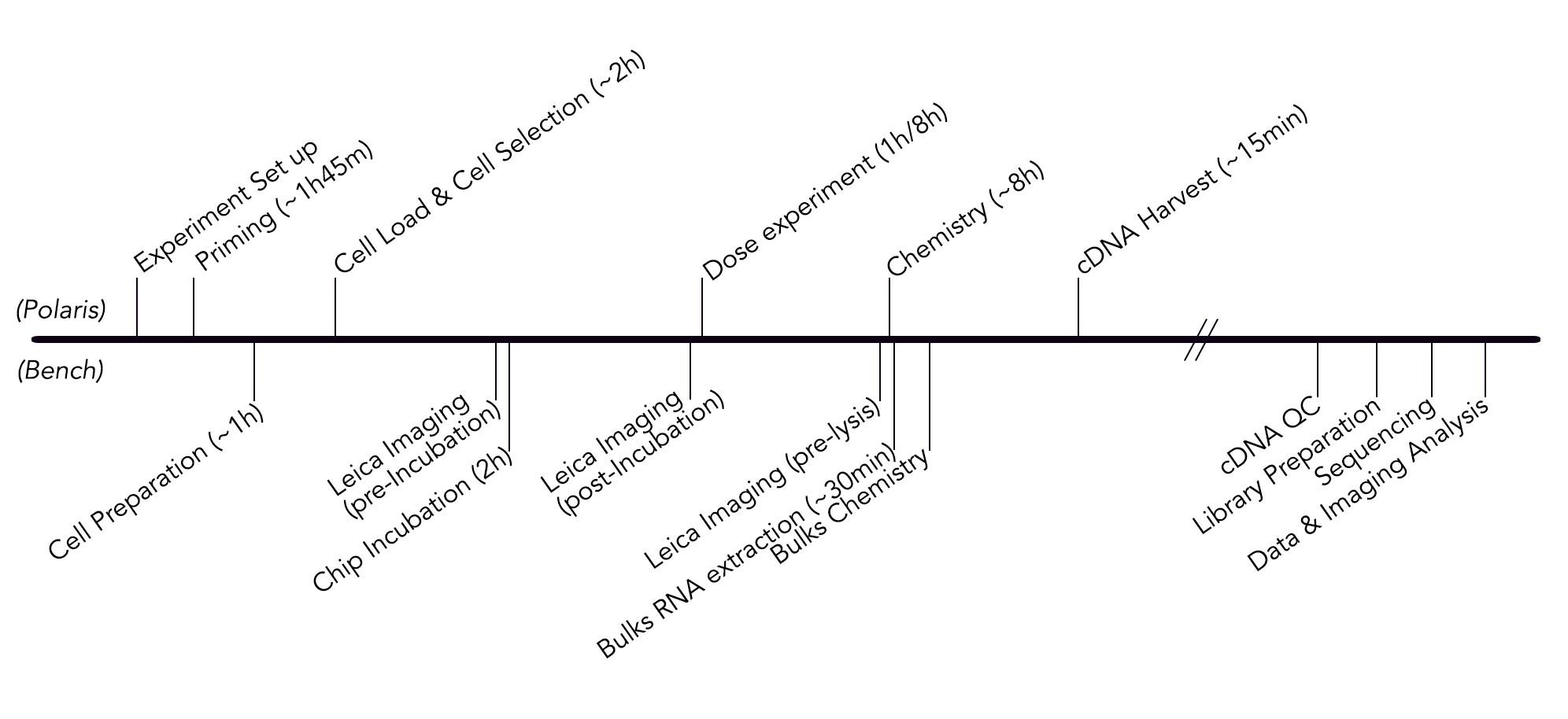


**B**


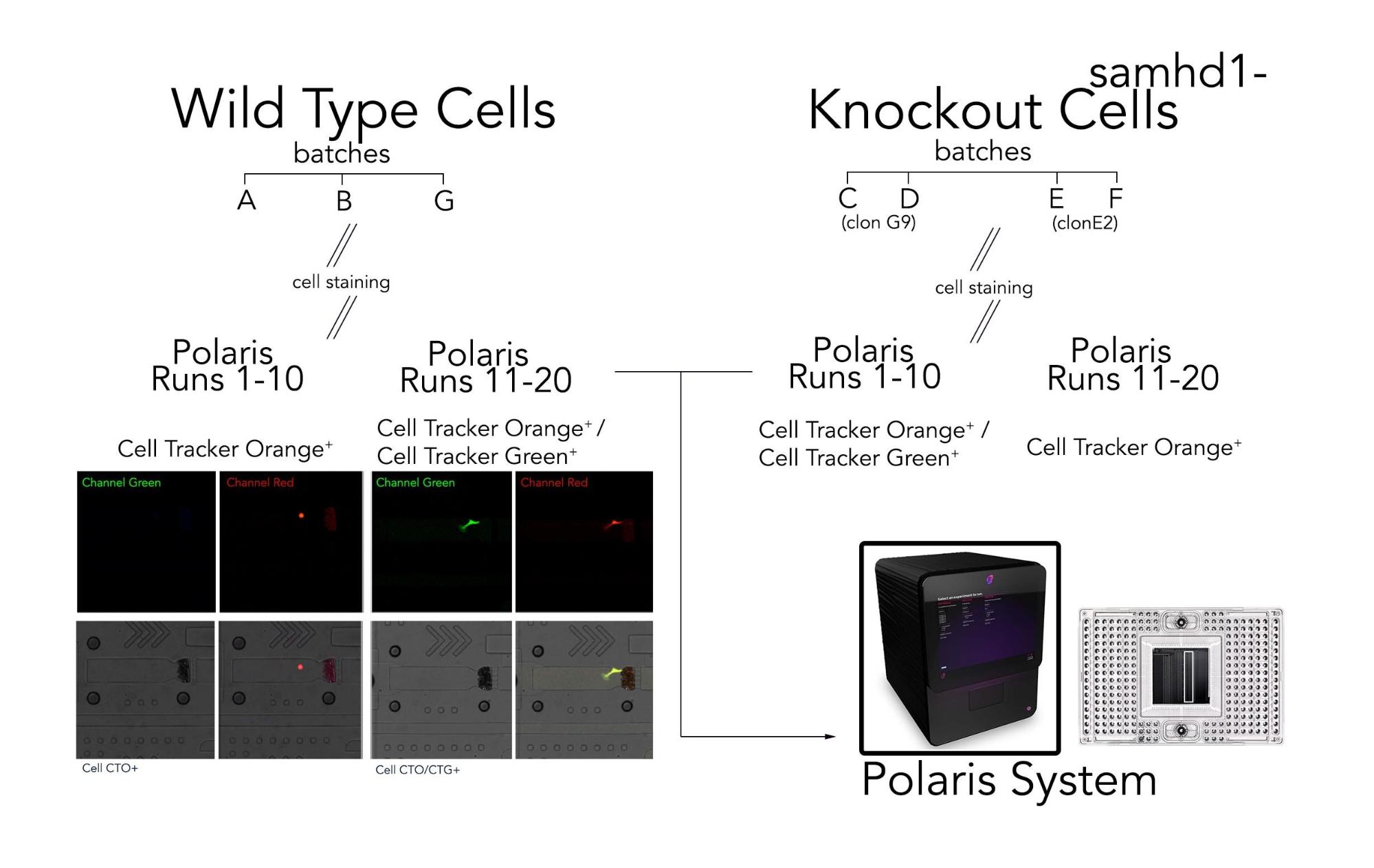


**C**


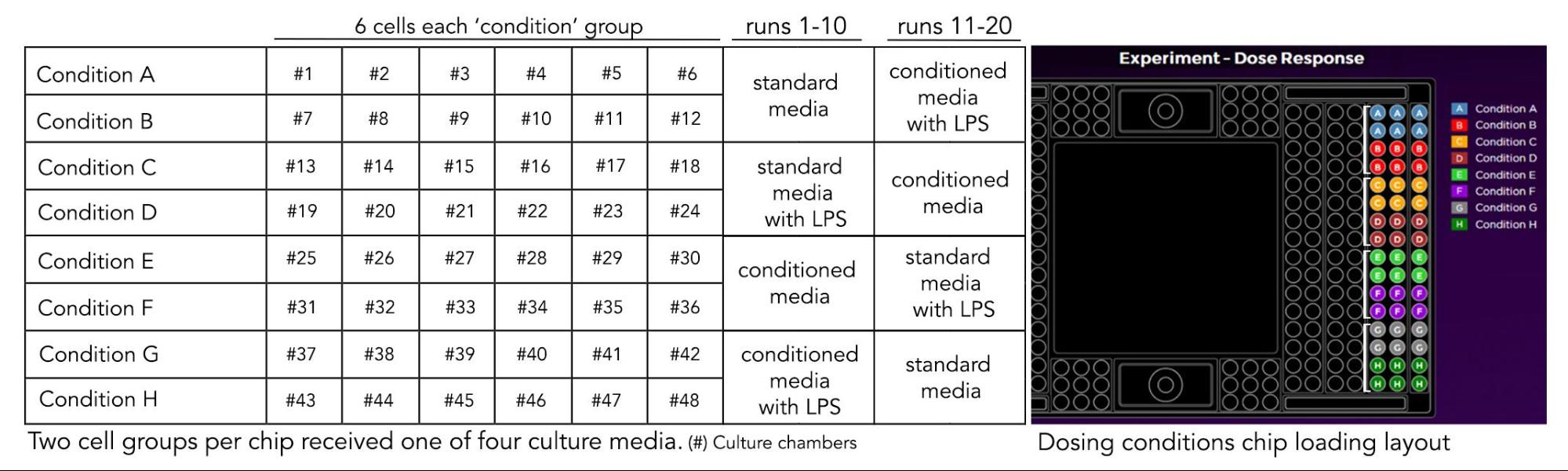


**D**


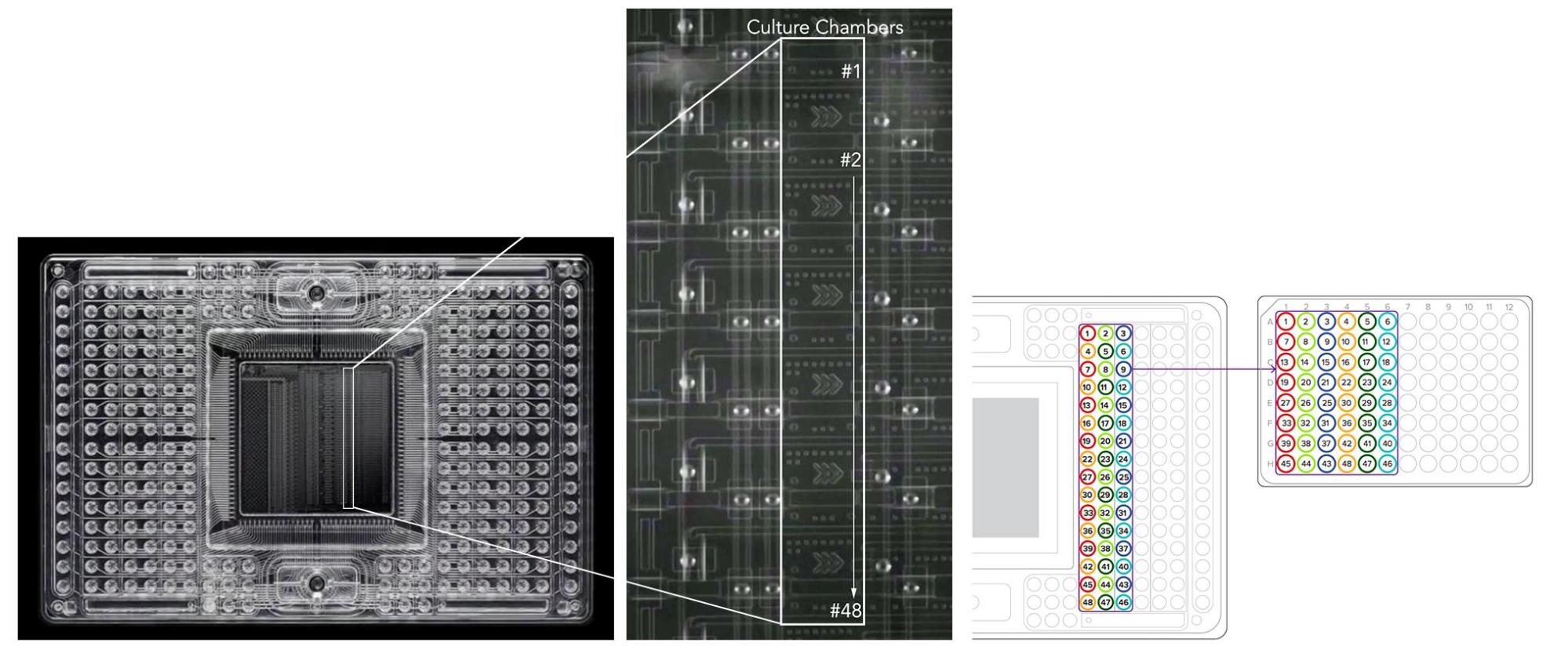


**Fig. S2.** Experimental overview. (A) Cell Staining. The Polaris used CellTracker Orange CMRA Dye to automatically identify and isolate cells. The two populations in each run were differentiated by the presence of CellTracker Green CMFDA Dye in either the wild-type or knockout cells. The arrangement of stains was switched between runs 1-10 and runs 11-20. (B) Overview of experimental timings. (C) Overview of dosing across experimental runs. In each run, two groups of 6 chambers each (12 cells total) were dosed with one of 4 media types (standard media, standard media with LPS, conditioned media and conditioned media with LPS). For runs 11 to 20, the original order of loading was reversed. Image shows IFC loading layout of dosing conditions (from Fluidigm documentation). (D) Relationship between culture chambers, IFC cell harvest wells and plate positions (from Fluidigm documentation).


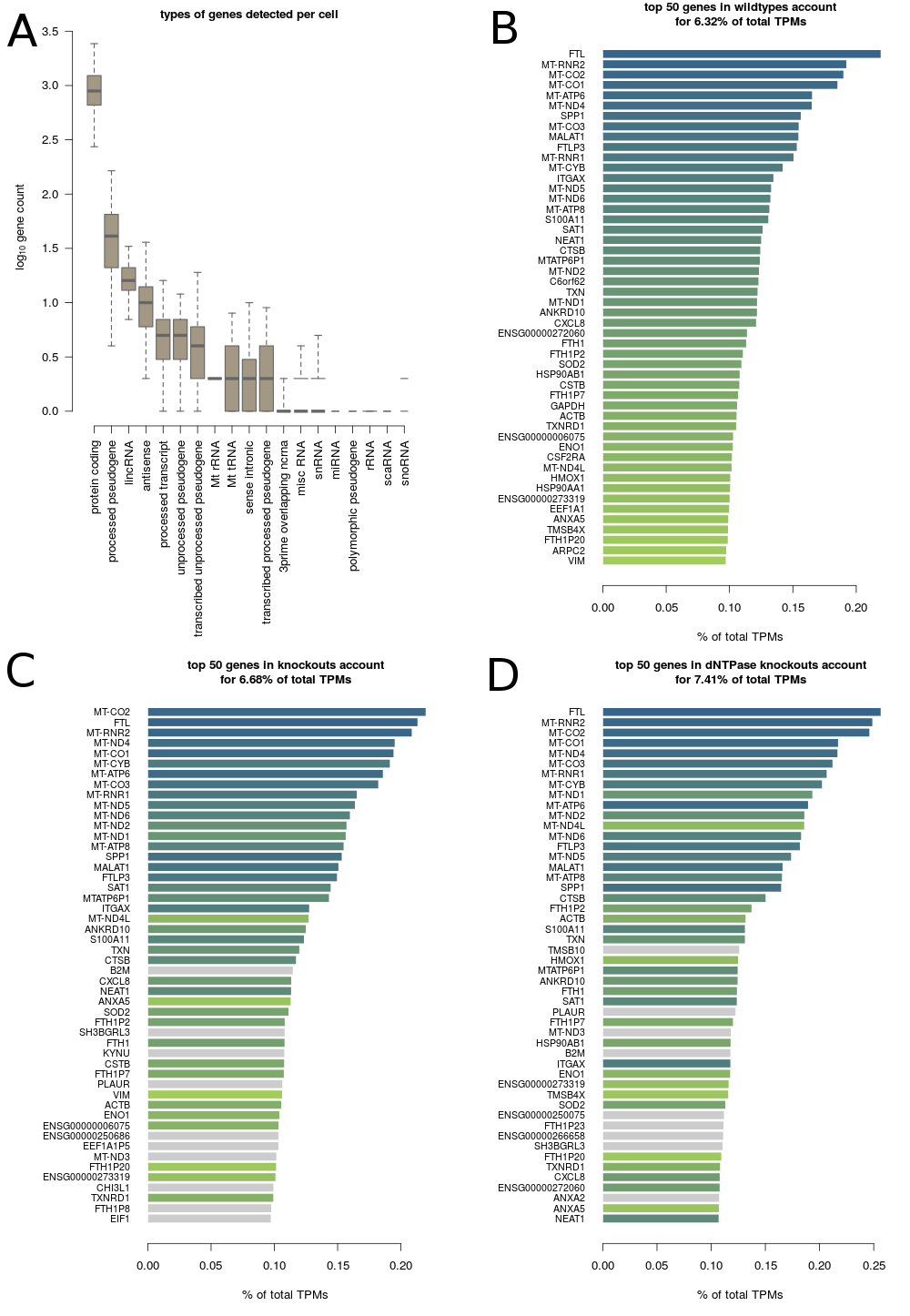


**Fig. S3.** (A) Summary of genes with detectable expression in 80% of replicates. (B-D) The top 50 gene plots are those for cells in conditioned media without LPS for eight hours. The coloured bars in the two types of knockouts are those top 50 genes shared with the wildtypes.


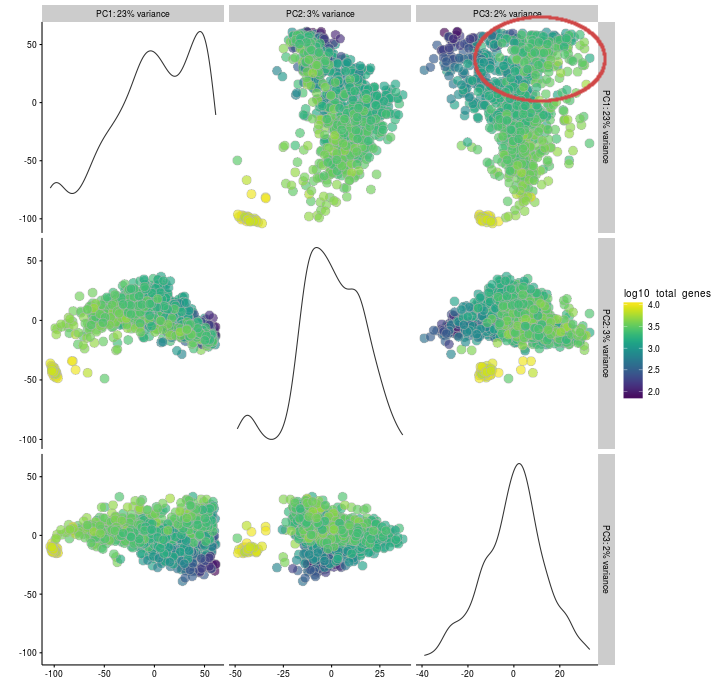


**Fig. S4.** PCA of macrophages and sequencing controls, demonstrating a gradient of total detected genes per cell in the first component and a second cluster of cells in the third component (circled in red).


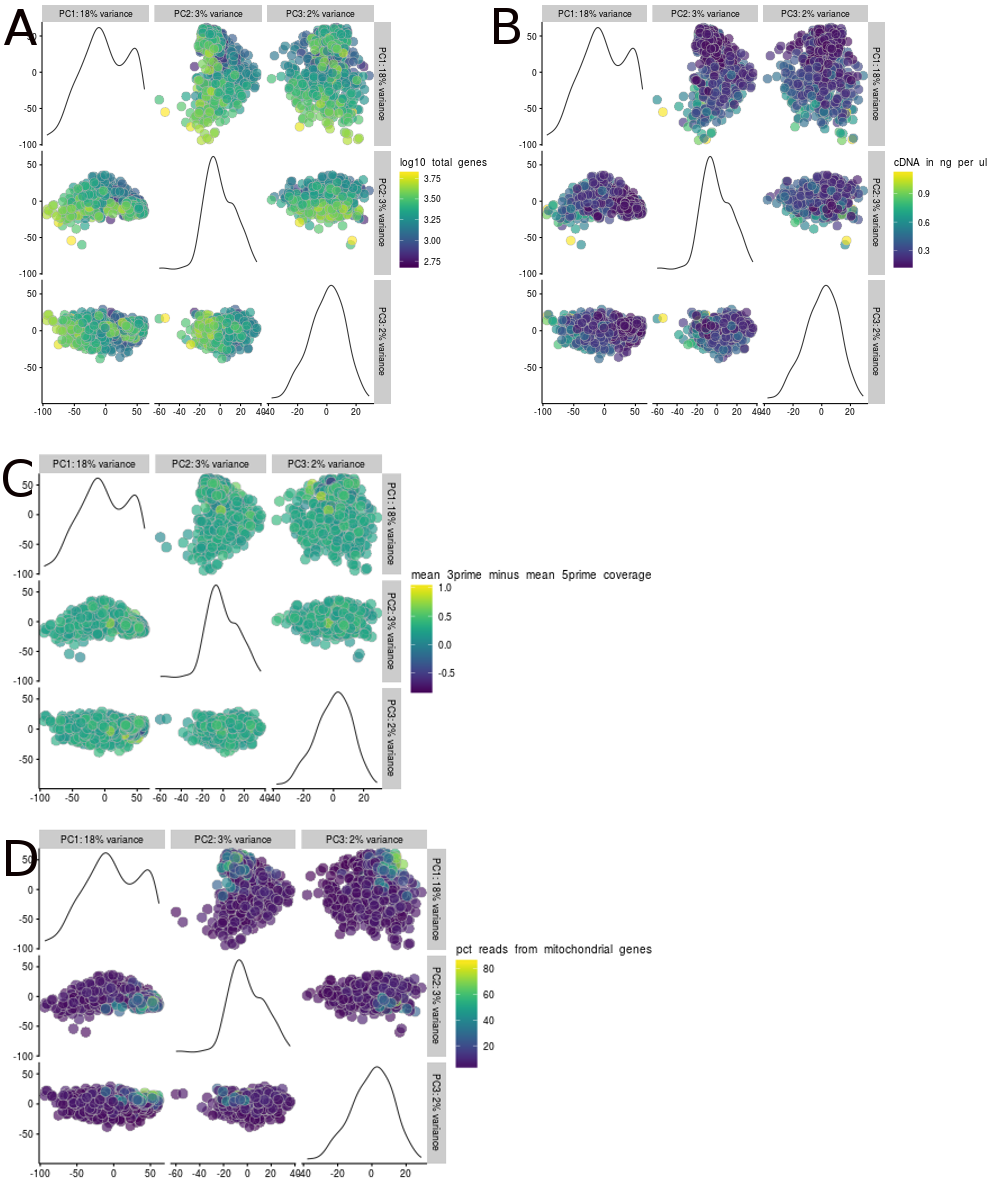


**Fig. S5.** Sequencing variables associated with the second cluster of cells.


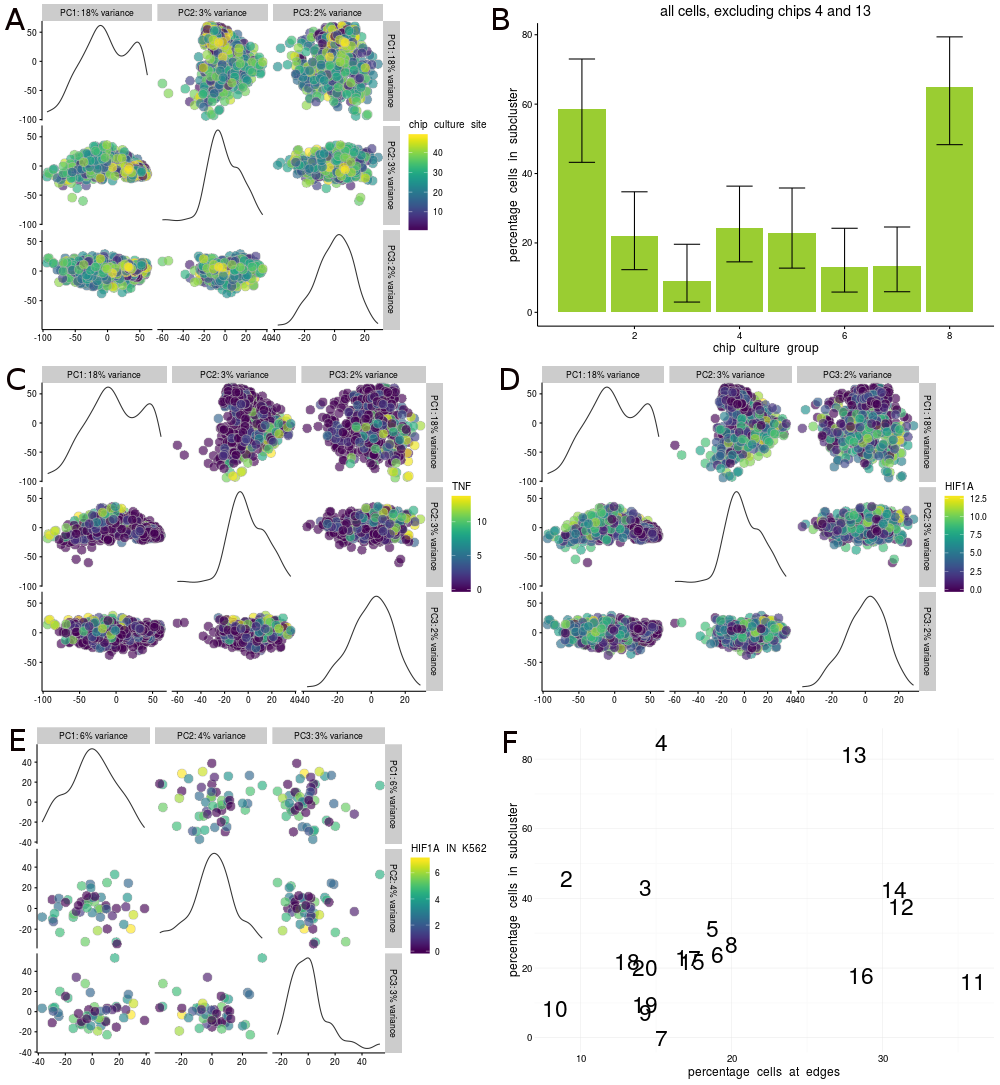


**Fig. S6.** Inter- and intra- chip effects. (A-D) Macrophages with low or high culture site numbers (chip culture groups 1 and 6) are enriched for in the second sub-cluster (Fig. S4), which demonstrates lower TNF and HIF1A expression. (E) Similar edge and HIF1A patterns were not found in reference K562 cells (F) Chips 4 and 13 enriched for cells in the second subcluster

**Fig. S7.
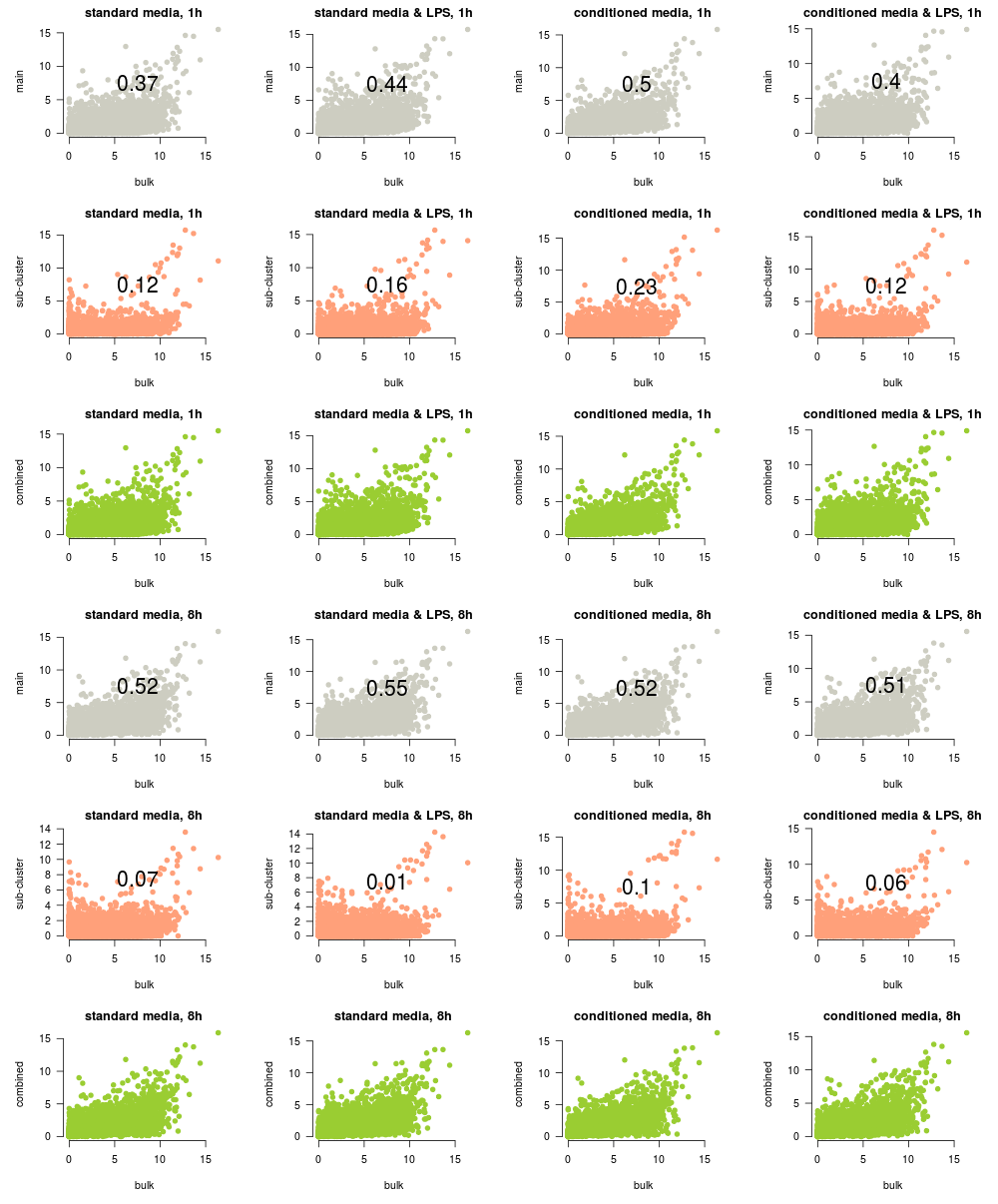
** Expression correlations of cells under different culture conditions with bulk controls. Scatterplots in grey represent the main cluster of cells observed with exploratory data analysis, while those in orange represent the observed sub-cluster. Spearman correlations are provided per plot, where it can be seen that the main cluster of cells demonstrate greatest bulk correlation at eight hours, while the sub-cluster correlation is poor. Green scatter plots are mixtures of cells from the two clusters that best correlate with bulks. In all cases a mixing proportion of greater than 99 percent from the main cluster was optimal, suggesting that the observed sub-cluster is unlikely to be representing cell populations off chip.


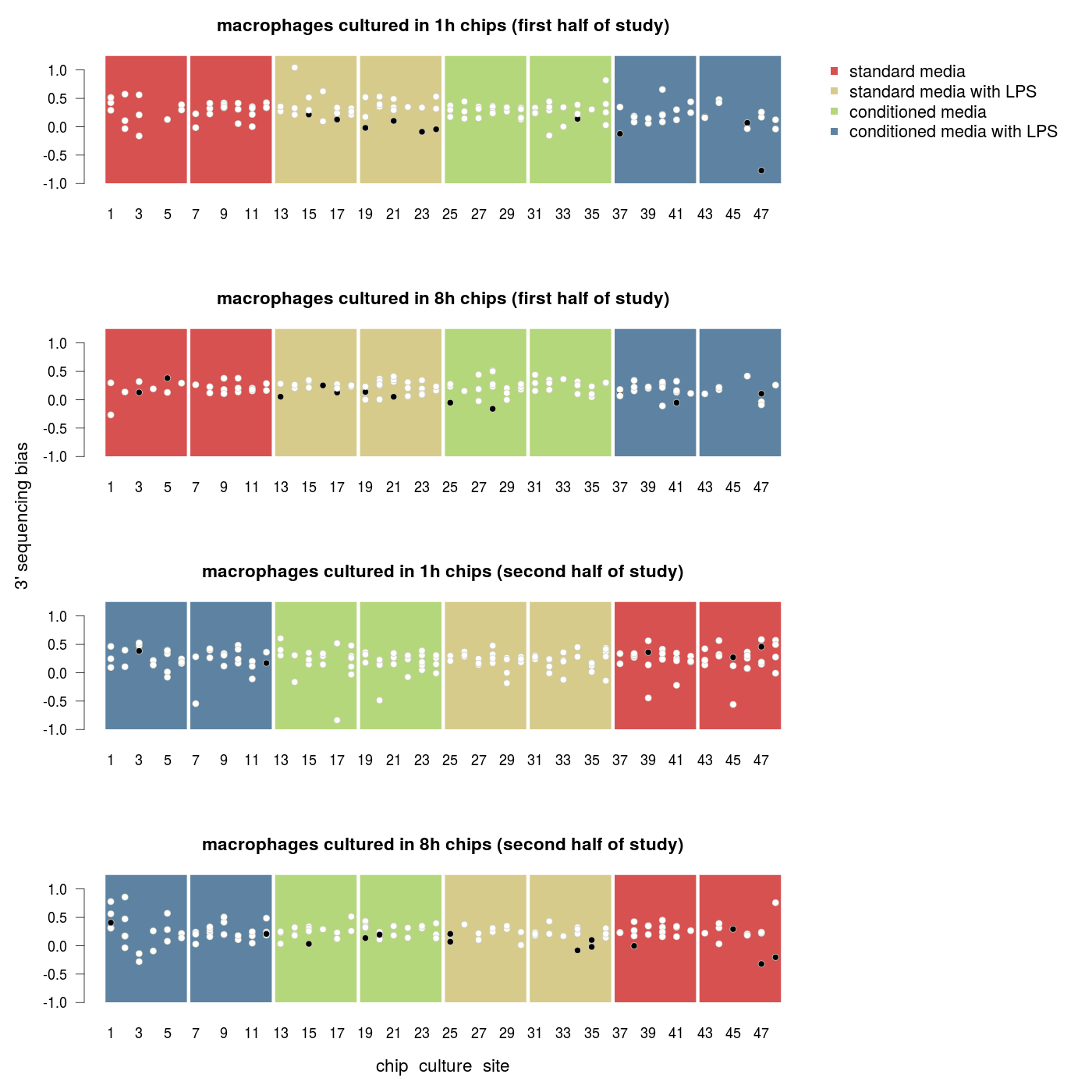


**Fig. S8.** Macrophages cultured towards the ends of chips demonstrated no change in sequencing coverage. Macrophages omitted from further analysis due to low starting material are plotted in black.


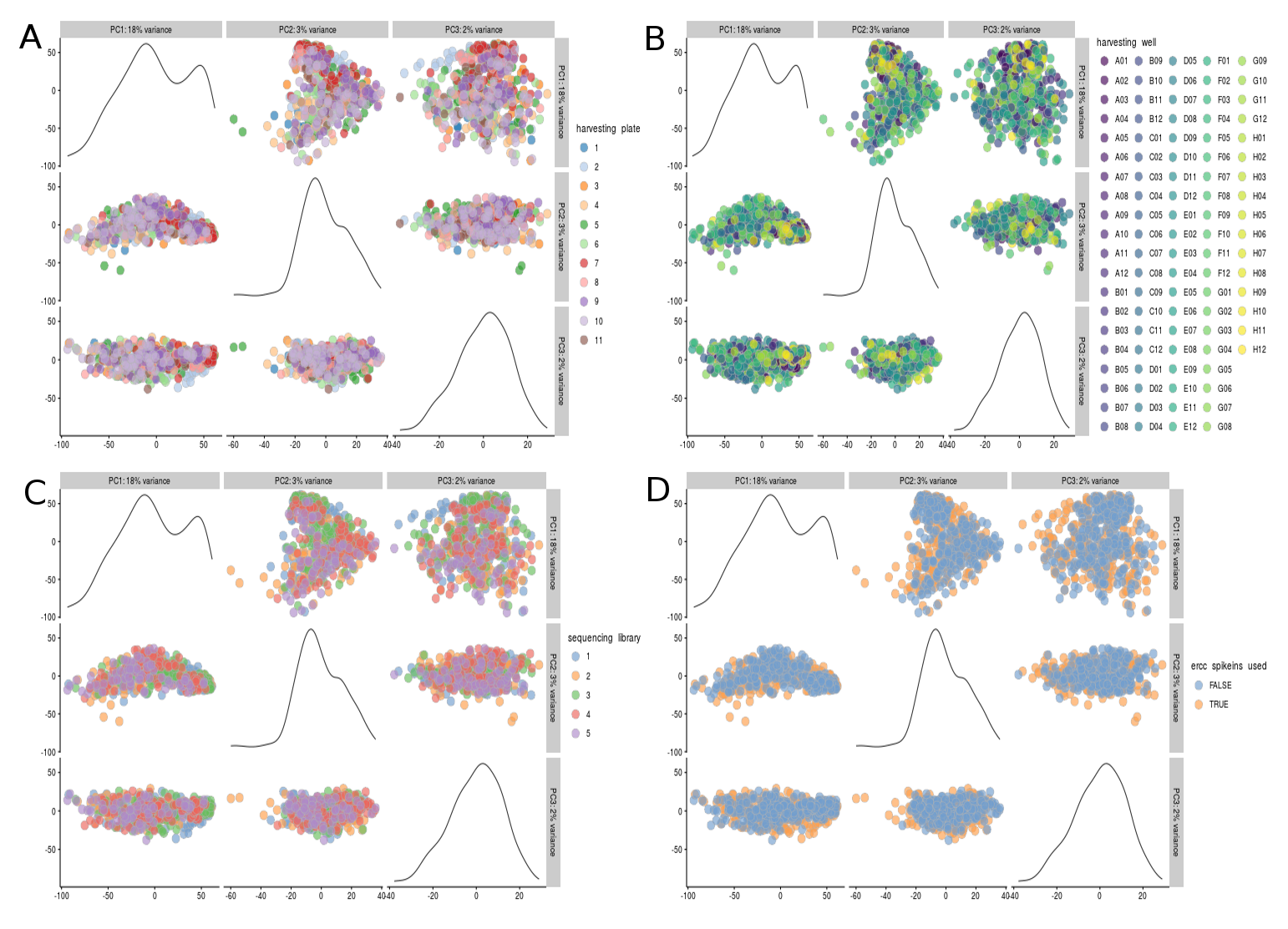


**Fig. S9.** Harvesting, spike-in and sequencing library effects (A&B) Harvesting plates do not demonstrate clustering, while harvesting wells are confounded with chip culture positions (C) sequencing and (D) spike-in batches demonstrate no association with the second sub-cluster of cells.


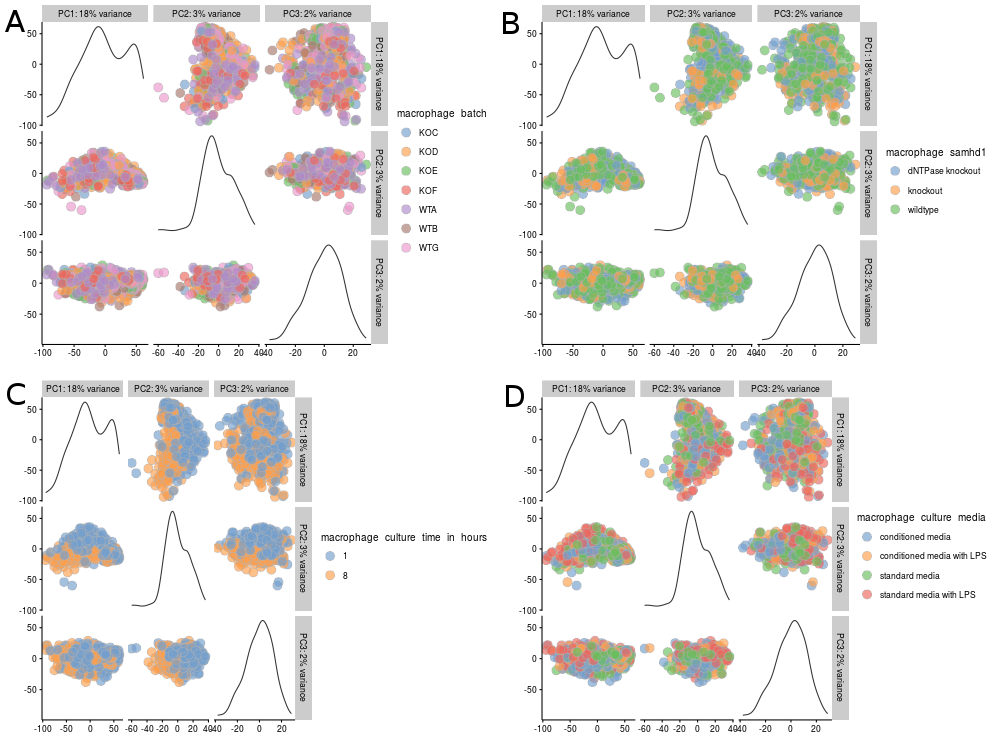


**Fig. S10.** Macrophage types and culture conditions. (A&B) No association between macrophage batch or knockout status and the second sub-cluster. (C) The separation of one hour and eight hour activated macrophages (D) No association between the culture media and the second sub-cluster.


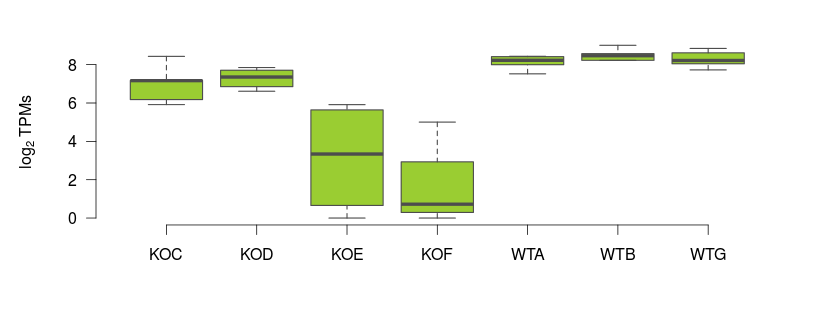


**Fig. S11.** SAMHD1 expression in the bulk controls for the various macrophage batches.


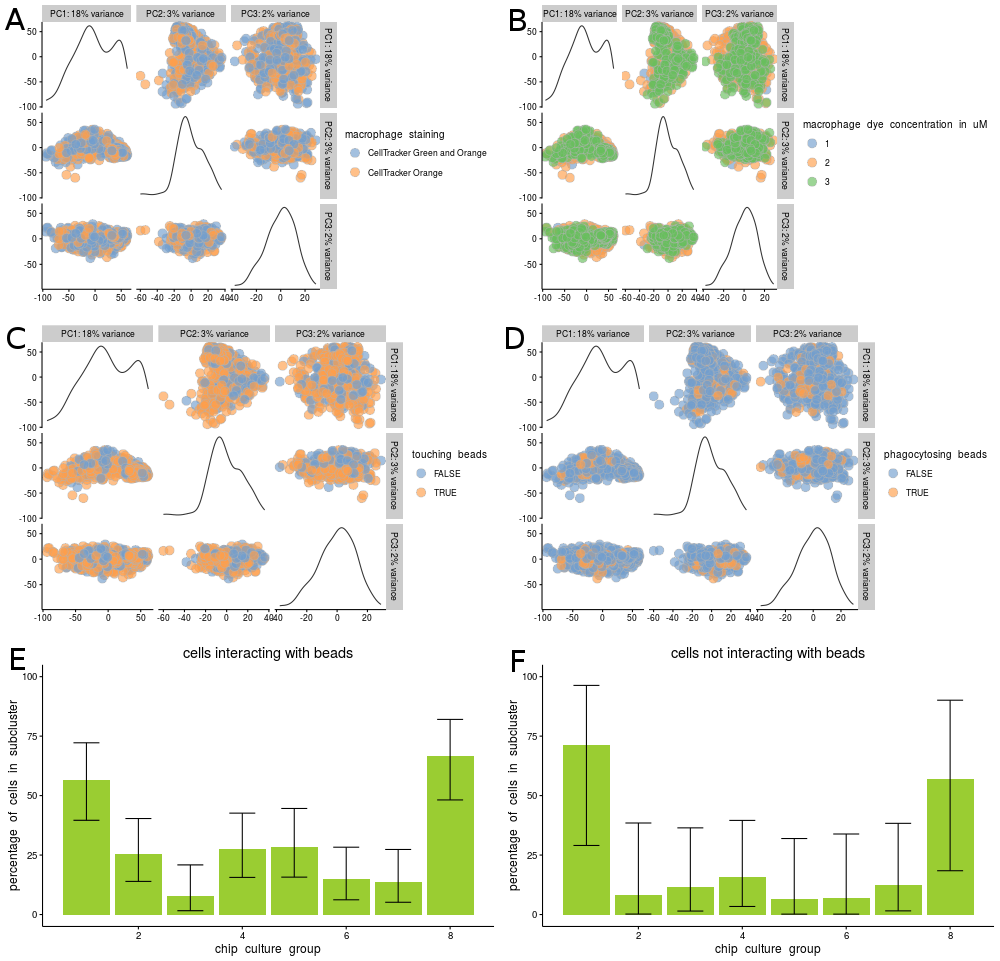


**Fig. S12.** Macrophage staining and bead associations. (A&B) No association was found between cell clustering and cell dyes. (C-F) While not obvious in these plots, macrophages interacting with beads (touching or phagocytosing beads) were more likely to occur in the sub-cluster, with cells cultured at the edges also more likely to touch beads beads. Associations are formally tested as part of the cluster analysis.


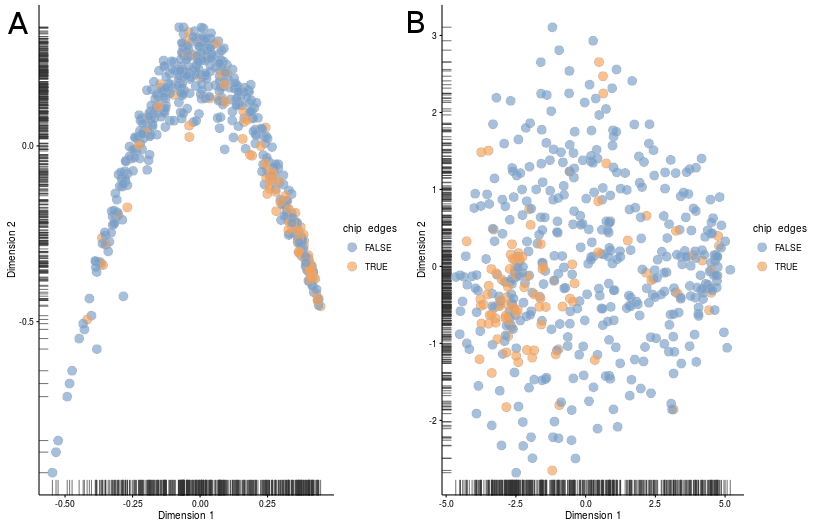


**Fig. S13.** (A) Diffusion Maps and (B) tSNE plots also capture the sub-cluster effect, shown here as the association with cells cultured towards the edges.


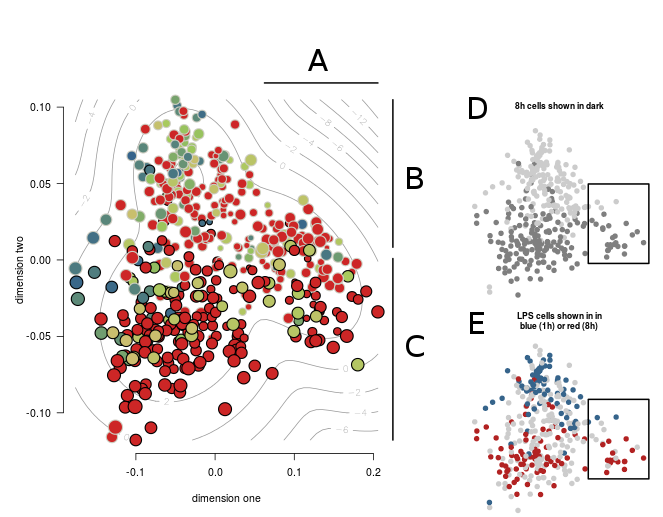


**Fig. S14.** MDS demonstrating (A) the sub-cluster and maintained separation of (B) one hour and (C) eight hour macrophages in both clusters of cells. Cells are sized by number of detected genes and coloured red to blue by increasing TNF expression. One hour cells have light margins, while eight hour cells have dark margins. (D-E) The same cells minus chips 4 and 13, and those cells cultured at the edges, demonstrating that the cluster (in the rectangles) is maintained.


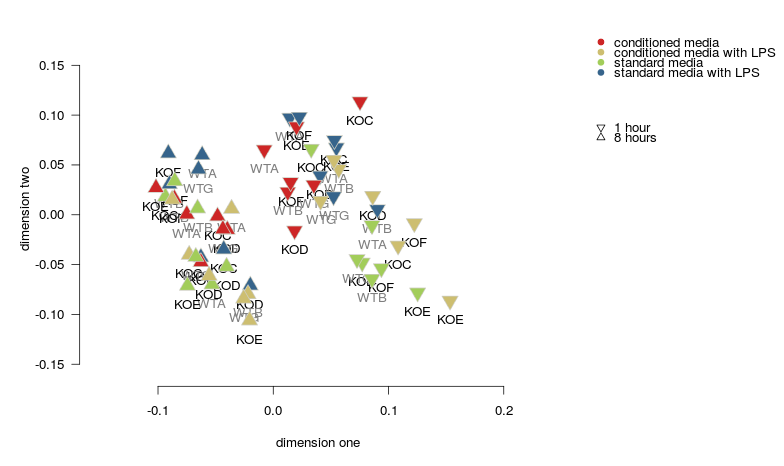


**Fig. S15.** MDS plot of the summarised experimental conditions demonstrating the clustering of one hour versus eight hour cells. Each condition shown is a summary of expression for that condition on a single chip.


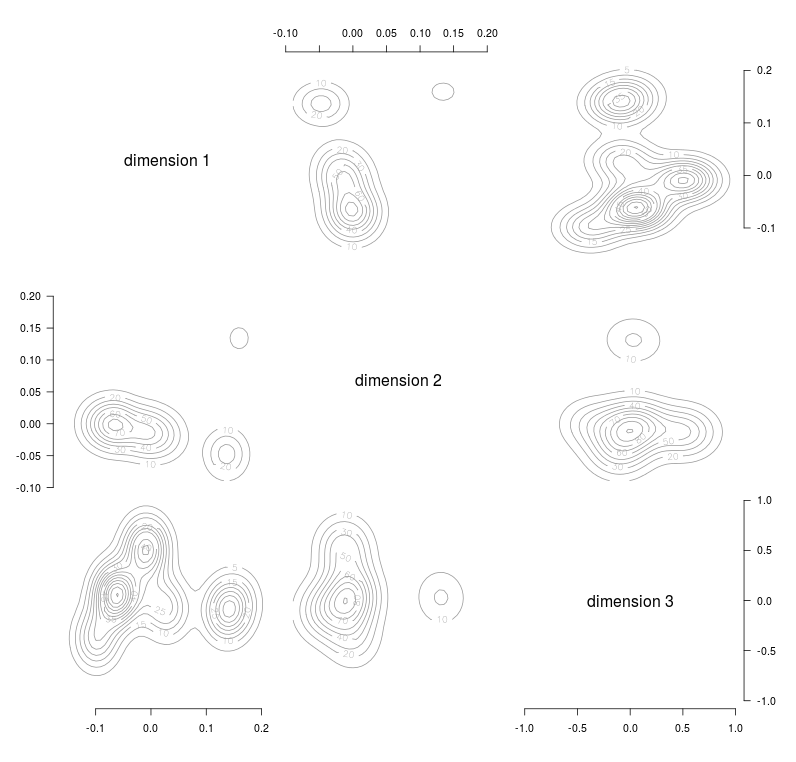


**Fig. S16.** Eight hour cell densities in the first five dimensions of a multidimensional scaling. Cell densities suggest multiple clusters but, as shown in Fig. S17, some of these are not reproducible across chips.


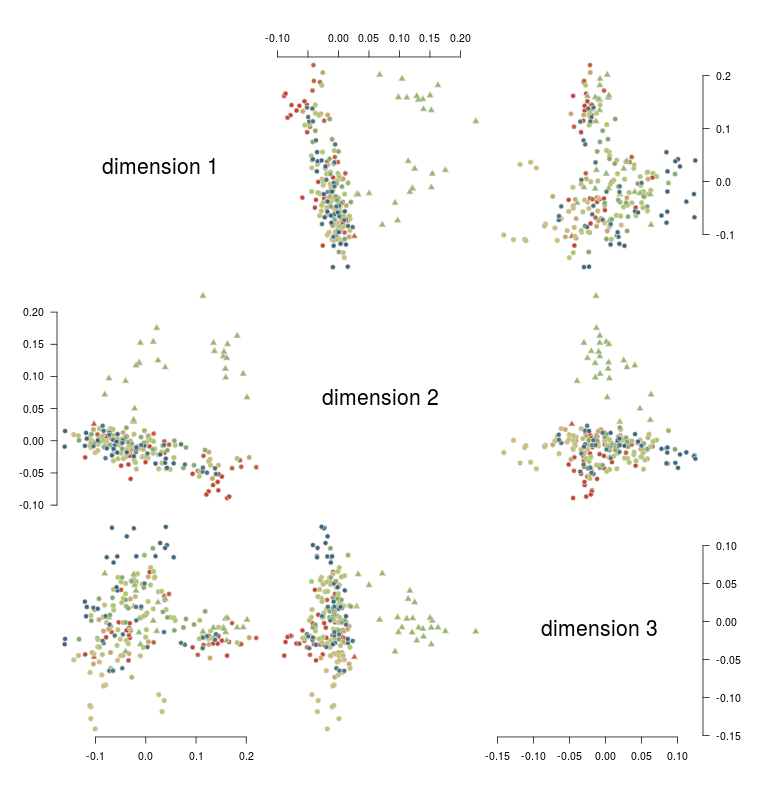


**Fig. S17.** Eight hour cells coloured by chips, with a group of cells (up arrows) unique to a single chip and so not considered in downstream analysis.


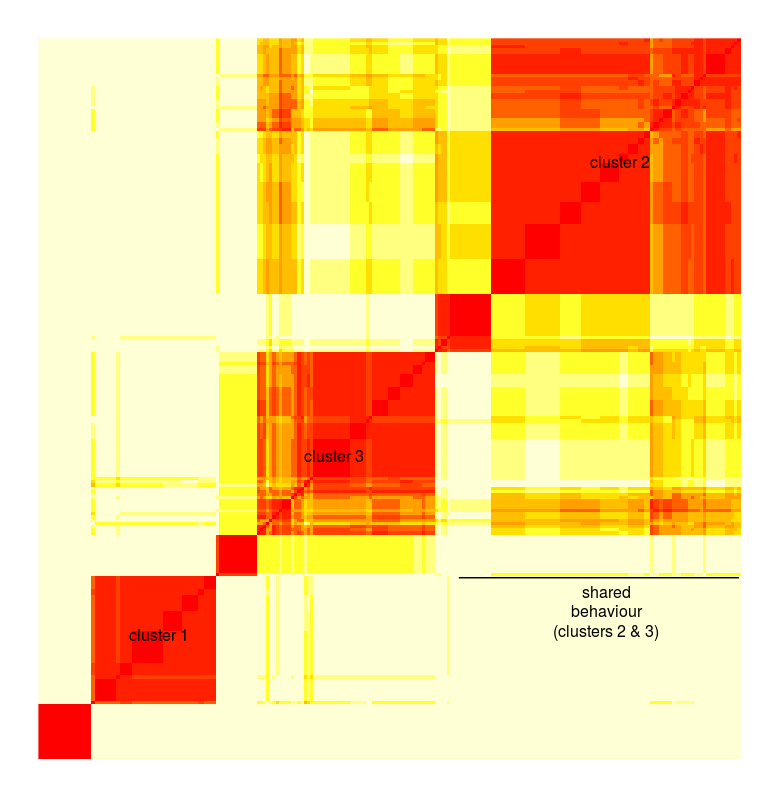


**Fig. S18.** Heatmap showing the clustering consensus matrix for cells at eight hours. The labelled red blocks form the centres of the clusters, with the dark yellow blocks indicating that cells from clusters 2 and 3 share clustering cells with the iterative subsampling.
